# A Review of the Phytochemistry, Molecular Docking, Pharmacology, Toxicology, Ethnopharmacology, Botany, and Clinical Studies of *Maytenus senegalensis* (Lam.) Excell

**DOI:** 10.3390/biom15020197

**Published:** 2025-01-30

**Authors:** Thanyani Emelton Ramadwa, Stephen Meddows-Taylor

**Affiliations:** Department of Life and Consumer Sciences, College of Agriculture and Environmental Sciences, Florida Campus, University of South Africa, Private Bag X6, Florida, Johannesburg 1710, South Africa; ramaate@unisa.ac.za

**Keywords:** *Maytenus senegalensis*, secondary metabolites, antiparasitic, antimycobacterial, clinical trials, pharmacology, phytochemistry, molecular docking

## Abstract

*Maytenus senegalensis* (Lam.) Excell, also known as *Gymnosporia senegalensis* (Lam.) Loes, is distributed particularly in savannah regions of tropical Africa. It is used to treat malaria, tuberculosis, rheumatism and diarrhea, amongst other afflictions. The objective of this comprehensive review is to summarize studies on phytochemistry, molecular docking, pharmacology, toxicology, ethnopharmacology, botany, and clinical trials of *M. senegalensis*. Data on *M. senegalensis* were collected using different databases such as Google Scholar, Science Direct, Web of Science, Scopus, SciFinder, Wiley Online, etc. This review showed that 118 biomolecules from different parts of *M. senegalensis* were identified. A total of 46 compounds were tested for antiplasmodial, anti-inflammatory, and antiproliferative activities, and some in vivo studies were carried out on mice. Isomintlactone (**31**), pristimerin (**24**), and jacareubin (**32**) were analyzed for molecular docking. The crude extracts and fractions had pharmacological activities, including antiparasitic, antimycobacterial, anti-inflammatory, antiviral, antiproliferative, and antidiabetic, while showing low toxicity in mice. Clinical trial studies on the safety and tolerability of *M. senegalensis* ethanol root bark extracts in male volunteers showed its potential immunomodulatory effects. Another trial specifically evaluated the electrocardiographic effects of *M. senegalensis* in adult volunteers and showed its advantageous cardiac profile by improving the overall safety profile.

## 1. Introduction

*Maytenus senegalensis* (Lam.) Excell (Celastraceae), alternatively known as *Gymnosporia senegalensis* (Lam.) Loes, is an important plant used in traditional medicine around the world. It is commonly known as red spikethorn or confetti spikethorn and is widely distributed, but most notably is in the savannah regions of tropical Africa, East Africa, the Middle East, the Arabian Peninsula, and India [1,2,3]. The species occupies a wide range of habitats, including forests, undergrowth areas, wooded grasslands, riverbanks, and swamp margins [1,3]. *M. senegalensis* grows normally up to 7–9 m tall [4].

The use of *M. senegalensis* in the treatment of a variety of diseases is widely documented in the literature and includes asthma, cough, diarrhea, pneumonia, tuberculosis, sore throat, earache, eye infections, helminth infection, malaria, dysmenorrhea, rheumatism, chronic wound healing, cancer, snakebites, dyspepsia, and venereal diseases [3,5,6,7,8,9,10,11,12,13,14,15,16,17,18,19]. Roots are used mainly in the preparation of traditional remedies, while leaves and stem bark are also used, but less frequently [2].

Several pure secondary metabolites have been isolated from *M. senegalensis* leaves, roots, root bark, and stem bark [10,20,21]. Two new methylated flavan-3-ol glucosides and a methylated proanthocyanidin were isolated from the methanol extract of the stem bark of *M. senegalensis* and reported for the first time from this plant [10]. Several studies have tentatively identified phytocompounds from the whole plant extract and root bark of *M. senegalensis* by gas chromatography and mass spectroscopy (GC–MS) and high-performance liquid chromatography-mass spectrometer equipped with an electrospray (ESI) interface (HPLC-ESI-MS^n^) [22,23,24,25]. Phytochemical constituents present in *M. senegalensis* include alkaloids [26], flavonoids [13], triterpenes, saponins, phenol, tannins, and glycosides [27,28]. Molecular docking studies of prostaglandin E synthase with compounds such as pristimerin, isomintlactone and jacareubin identified from *M. senegalensis* have been reported [24].

In vitro antiparasitic, anti-inflammatory, antiviral, antimicrobial, antiproliferative, anti-sickling, antidiabetic, antioxidant, and antiviral activities of *M. senegalensis* on different parts of the plant fractions have been observed, and several isolated compounds have been reported [9,10,29,30,31]. In vivo studies on animal models through oral administration have demonstrated tolerability and low toxicity of *M. senegalensis* [14,32]. The first clinical study assessing the safety and tolerability of *M. senegalensis*, a herbal treatment for malaria, in healthy adult male volunteers has been published [33,34]. Despite many published studies on the genus *Maytenus*, *M. senegalensis* has not been thoroughly reviewed. Moreover, in ethnopharmacological research, only a few plant species have been validated to the clinical trial stage. Owing to the extensive research conducted on the identification and isolation of biologically active compounds, molecular docking studies, pharmacological activities, ethnopharmacology, and in vitro and in vivo toxicological studies and clinical trials on this plant species, the purpose of this review is to examine the phytochemistry, molecular docking, pharmacological studies, toxicology, ethnopharmacology, and clinical studies of various parts of *M. senegalensis*.

## 2. Materials and Methods

The information gathered from several internet search engines served as the foundation for the search. Several scientific search engines were used to collect data on the phytochemistry, molecular docking, ethnopharmacology, botany, medicinal uses, phytochemical analysis, pharmacological activity, biological activity, and pre-clinical and clinical studies of *M. senegalensis*, including SpringerLink, Wiley Online, PubMed, ScienceDirect, Scopus, Google Scholar, Web of Science, and SciFinder. After that, the gathered data were separated into each field for analysis in this review study.

Several keywords linked to *M. senegalensis* or *Gymnosporia senegalensis* were used for this analysis, including *M. senegalensis* or *G. senegalensis*, *M. senegalensis* or *G. senegalensis* extracts, *M. senegalensis* or *G. senegalensis* phytochemistry, *M. senegalensis* or *G. senegalensis* molecular docking, botany of *M. senegalensis* or *G. senegalensis*, ethnopharmacology of *M. senegalensis* or *G. senegalensis*, pharmacological activities of *M. senegalensis* or *G. senegalensis*, preclinical studies of *M. senegalensis* or *G. senegalensis* and clinical studies of *M. senegalensis* or *G. senegalensis*.

Inclusion Criteria

- Access to full-text English articles or articles in any other language that can be translated into English.
- All articles that contained the keywords were included, regardless of the publication date.
- Peer-reviewed publications were given preference.
- For this review, only publications from 1962 to 2024 were included.
- Out of the 140 published papers that were first examined, 67 studies are included in the study.

Exclusion Criteria

- Articles that were not published in English or could not be translated into English were excluded.
- Articles containing details about the plant, but beyond the purview of this review were excluded.

## 3. Results and Discussion

### 3.1. Botanical Characterization and Distribution

*M. senegalensis*, also known as *G. senegalensis*, is a bright, white-flowered, sweet-scented indigenous shrub or small tree, often with wine-red branches, belonging to the family of Celastraceae. This plant is common and scattered throughout tropical Africa and South Africa, from Senegal to Eritrea, southern Africa, and Madagascar. *M. senegalensis* also occurs on any soil in the Guinean and Sudanese savannahs [35,36]. Its distribution in the southern part of Africa ranges from KwaZulu-Natal in South Africa to Namibia and northwards throughout Africa, Europe, and the central and southern parts of Asia. It is also found in Madagascar, Mauritius, and the Seychelles. Often in groups of impenetrable bushes, it is a small tree up to 8 m in height. It can be recognized by its pale leathery obovate leaves, its grey scaly bark with red branches, and the flowers during the dry season after the plant has come into leaf [35,36].

**Figure 1:**
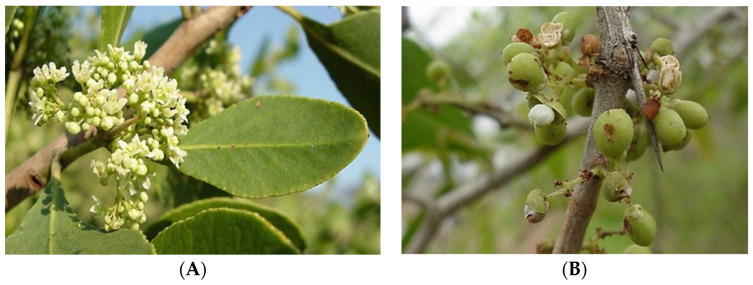
*Maytenus senegalensis* leaves and flower (**A**), and bark and fruits (**B**), courtesy of https://pza.sanbi.org/gymnosporia-senegalensis (accessed on the 26 September 2024).

The branches have thin spines up to 70 mm long and are frequently wine-red in color. Clustered, elliptic to obovate, 20–125 × 4–80 mm in size, leathery, pale blue-green, with a whitish bloom, and hairless, the leaves have many regular, rounded teeth set closely apart on the border. As shown in Figure 1, the flowers are tiny, cream-colored or greenish-white, have a pleasant aroma, and are produced in axillary heads with many flowers in the winter and spring (June to October). The fruit is a round capsule with a diameter of around 5 mm. It is smooth, pinkish to reddish, and has reddish-brown seeds that are partially covered by a pinkish or white aril [37,38,39].

### 3.2. Ethnopharmacological Uses

The leaves, bark, seeds, and roots of *M. senegalensis* are used to treat a wide range of diseases in different African countries and other parts of the world, as presented in Table 1. This plant has been reported to treat fever and diarrhea [40,41,42], in addition to being used for abscesses [43]. The brewed roots are used for tooth pain, wound skinning, and gonorrhea while the leaves are recommended for ocular illnesses, tooth pain, stomatitis, and gingivitis, and as anti-bilharzial and anti-ulcerous gastric agents [44]. In the southern part of Africa, *M. senegalensis* roots are used traditionally to treat and prevent persistent cough, tuberculosis, and sexually transmitted infections in Botswana [45] and to treat schistosomiasis in South Africa and Zimbabwe [46]. In Zimbabwe, the roots and leaves of *M. senegalensis* are used for various respiratory ailments, including pneumonia and tuberculosis [47]. In Zambia, the leaves are crushed, soaked in water, and drunk to treat tuberculosis [48]. In Senegal, roots are used to treat malaria [40]. In Kenya, roots are used to treat chest pain, rheumatism, snakebites, diarrhea, and fever. Leaves are used to treat eye infections [49]. The root bark is used to treat malaria in Kenya. The preparation of the plant treatment is by hot water decoction in soup [50]. The leaves, bark, and root of *M. senegalensis* are used to manage opportunistic infections associated with HIV/AIDS and to treat bacterial (syphilis) and fungal (oral candidiasis) infections in Uganda. The reported methods of preparation for the leaves, bark, and root are boiling or pounding, or with the addition to water and being used for bathing [51]. According to a recent study carried out in eastern Uganda, the roots are used to treat diabetes mellitus [52]. The bark and leaves are used to treat non-small cell lung cancer in Ethiopia. The bark is crushed, boiled, and filtered, and a cup of the mixture is served while the leaves are applied to the affected region as a paste [3]. Fresh/dry *M. senegalensis* powdered seeds with water or butter are taken with coffee or tea for five days to treat or prevent epilepsy in the same country. Furthermore, fresh/dry powdered seeds are taken in combination with *Ocimum lamiifolium* seeds with coffee as a drink to treat headaches [53]. According to Tewelde and Mesfin [54], the bark is used to treat stomach pain in Ethiopia, where the bark is prepared by crushing, soaking, and thereafter drinking it. The stem bark decoction and infusion are used to treat diarrhea in Togo [55]. In Burkina Faso, *M. senegalensis* bark, roots, and leaves are used to treat oral diseases such as toothache, gingivitis, and sores. The bark and leaves are dried separately, pounded, and strained. The fine powder obtained is then stored in a bottle or other airtight container and used for at least 5 years. In cases of gingivitis or sores, a small amount of the powder is rubbed onto the painful site twice a day (morning and evening) until recovery, generally for about 7–10 days. A decoction of roots and leaves is prepared and stored only for the treatment period of 2–3 days. It is used for inhalations and mouthwashes, generally in the morning on waking and after the evening meal to relieve pain. A bark decoction is also used as a gargle until recovery from pain. Childhood signs of cancrum oris (noma) or necrotizing gingivitis are serious, with heavily bleeding gums and very bad breath. In such cases, the dried leaves are pounded, and the powder is applied to the affected sites to immediately stop the bleeding and cure the sores [56]. The leaves and root decoction are also reportedly used to treat malaria, diarrhea, dental pain, and headaches. The mode of administration is by bathing and gargling. This plant is also reported to be used to treat dysentery, ulcers, diarrhea, stomachache, headache, tiredness, cold, insomnia, fatigue, dental infections, and eye infections in the same country [57]. In Nigeria, *M. senegalensis* roots are used to treat stomachaches and snakebites. The plant is prepared by peeling the root bark and thereafter boiling the roots and allowing them to cool. The dosage or mode of administration is by drinking a cup a day until the aches are no longer present. Regarding snake bites, the mode of administration is by drinking a cup 3 times a day for 7 days. The leaves and bark are used to treat dysentery. The plant is prepared by boiling plant parts and allowing them to cool. Thereafter, a cup is drunk 2 times a day for 3 days. *M. senegalensis* roots are used to treat amoebic dysentery and yellow fever. The roots are prepared by boiling and thereafter, drinking 100 mL, three times daily for a week for amoebic dysentery, while for yellow fever, the patients drink a cup, twice a day, and are advised not to eat palm oil. The bark is used to treat ulcers and wounds. The remedy is prepared by boiling bark, allowing it to cool, and drinking a cup 2 times a day for 5 days [58]. In Sudan, the stem bark of *M. senegalensis* is widely used to treat tumors, dysentery, and snakebites [10]. *M. senegalensis* roots are used in the treatment of menorrhagia and leucorrhea in India. The mode of administration/preparation is as a juice [59].

### 3.3. Phytochemical Analysis

Qualitative phytochemical tests were carried out on *M. senegalensis* methanol and ethyl acetate root bark extracts, and it was established that the methanol extract contained tannins, saponins, alkaloids, flavonoids, anthraquinones, terpenoids, coumarins, phlobotanins, and sterols, while the ethyl acetate extract contained only tannins, saponins, and terpenoids [61]. Kpoyizoun et al. [62] screened the hydro-ethanolic extract for qualitative phytochemical analysis using the Folin–Ciocalteu method and determined the total flavonoids and total phenols present in the hydroethanolic extract. They revealed that the chemical groups present in the hydro-ethanolic extract include flavonoids, tannins, saponins, carbohydrates, and reducing compounds. The quantity of total phenols and total flavonoids in the hydroethanolic extract was measured using gallic acid and quercetin. The amount of total phenol was 45.26 mg of gallic acid equivalent per gram of extract, and the amount of flavonoid was 29.59 mg of quercetin equivalent per gram. Sanda et al. [63] conducted qualitative phytochemical screening of the ethanol leaf extracts. The result revealed the presence of both primary and secondary metabolites, i.e., carbohydrates, cardenolides, cardiac glycosides, flavonoids, saponins, tannins, and terpenoids. Ndako et al. [24] determined the quantitative phytochemical analysis of the methanol extract from *G. senegalensis* root bark. The Folin–Ciocalteu reagent standard protocol was used to determine the total phenol content. A spectrophotometer method, based on the formation of a flavonoid–aluminum complex that absorbs maximally at 415 nm, was used to determine the total flavonoid content. The Folin Denis reagent was used to determine the tannin (https://www.sciencedirect.com/topics/pharmacology-toxicology-and-pharmaceutical-science/tannin, accessed on 21 January 2025) content, while a gravimetric method of the American Oil Chemist Society (AOAC) was used for saponin in the samples. The total alkaloids were quantitatively estimated spectrophotometrically at 565 nm using vincristine (https://www.sciencedirect.com/topics/pharmacology-toxicology-and-pharmaceutical-science/vincristine, accessed on 21 January 2025) as a reference standard. The study revealed that the extract contained alkaloids, flavonoids, phenols, tannins, glycosides, terpenes, saponins, and anthraquinones. Flavonoids (134.12 ± 0.35 µg/mg) were the most abundant (*p* < 0.05) metabolites, followed by alkaloids (85.35 ± 0.23 µg/mg), while the quantity of saponins, phenols, and the tannins content of the plant were 69.59 ± 0.35, 47.40 ± 1.34, and 32.34 ± 0.78 µg/mg, respectively. The quantitative phytochemical contents of phenols, flavonoids, flavanols, tannins, and saponin were assessed from various fractions extracted from the leaves, stem, and bark of *G. senegalensis* using standards like rutin, gallic acid, quercetin, tannic acid, and saponin quillaja [64]. The highest total phenol concentration was found in the stem chloroform extract at 97.7 ± 0.02 mg GAE/g. The amounts of total flavonoids and flavonols in the aqueous extract were 97.1 ± 0.03 mg QE/g and 96.7 ± 0.07 mg RE/g, respectively. The total saponin content in the stem methanol extract was 79.1 ± 0.06 mg SQE/g, whereas the total tannin concentration in the leaf ethyl acetate extract was 97.5 ± 0.01 mg TAE/g.

### 3.4. Isolated or Tentatively Identified Compounds

Several studies have reported the isolation or tentatively identified secondary metabolites from different parts of *M. senegalensis*, as shown in Table 2. A total of 118 compounds have been identified from the *M. senegalensis*. Hussein et al. [65] isolated for the first time new methylated flavan-3-ol glucosides and methylated proanthocyanidin and other known compounds from the methanol extract of the stem bark of *M. senegalensis*. All the compounds that were isolated were (−)-4′-methylepigallocatechin (**1**), (−)-4″-methylepigallocatechin 5-O-β-glucopyranoside (**2**), (−)-epigallocatechin (**3**), (+)-4″-methylgallocatechin 3″-O-β-glucopyranoside (**4**), epicatechin (4β→8) epigallocatechin (**5**), (−)-epicatechin (4β→4) (−)-4 0-methyl epigallocatechin (**6**), epicatechin (4β→8) epicatechin (procyanidin B-2) (**7**), and phloroglucinol 1-O-β-D-glucopyranoside (**8**). Lindsey et al. [20] reported the isolation of the antibacterial compound 3-oxo-friedelan-20α-oic acid (**9**), more commonly known as maytenoic acid, from the root bark of *M. senegalensis*. In another study by Okoye et al. [16], bioactivity-guided fractionation of the methanol leaf extract of *M. senegalensis* led to the isolation of 11 compounds using column chromatography. A new compound, phenyldilactone, maysedilactone (**10**) together with other known compounds **2**–**11** were isolated, i.e., 9,10-dihydroxy-4,7-megastigmadien-3-one (**11**), (−) epicathechin (**12**), (+) gallocathechin (13), (−) epigallocathechin (3), procyanidin B-2 (14), 2,3-dihydrokaempferol 3-O-β-D-glucopyranoside (**15**), quercetin 3-O-β-D-glucopyranoside (**16**), kaempferol 3-O-β-D-xylopyranoside (**17**), quercetin 3-O-β-D-xylopyranoside (**18**) and 3,5-dimethylgallate (**19**). Four secondary metabolites, namely maytenoic acid (**9**), lupenone (**20**), β-amyrin (**21**), and β-sitosterol (**22**) were isolated from *M. senegalensis* roots using flash chromatography [21]. Jigam et al. [23] used gas chromatography coupled with mass spectrometry (GC-MS) to identify the secondary metabolites from the purified leaf fraction of *M. senegalensis*. Secondary metabolites from the fraction were identified as 3-hydroxy-20(29)-lupen-28-ol (12.95%) (**23**), 20α)-3-hydroxy-2-oxo-24-nor-friedela-1(10),3,5,7-tetraen-carboxylic acid-(29)-methylester (6.0%) (**24**), 2(4H)-Benzofuranone, 5,6,7,7a-tetrahydro- (7.0%) (**25**), and phytol (1.44%) (**26**). Other compounds identified in minute amounts include n-hexadecanoic acid (0.207%) (**27**), 9,12-octadecadienoic acid, methyl ester (1.67%) (**28**), cis-vaccenic acid (0.4.90%) (**29**), and 6-methyl-cyclodec-5-enol (0.66%) (**30**).

Ndako et al. [24] used GC–MS to identify the presence of compounds from the crude methanol extract and the polar fraction from *G. senegalensis* root bark. GC–MS analysis revealed the presence of nine secondary metabolites, of which four were polar compounds: 5,6,7,7a-tetrahydro-2(4H)-benzofuranone (isomintlactone) (**31**), ((20a)-3 hydroxy-2-oxo-24-nor-friedela 1(10),3,5,7-tetraen (pristimerin) (**24**), 2H,6H-pyrano[3,2-b] xanthen-6-one (jacareubin) (**32**), and 5,7,3′-trihydroxy-6,4′-dimethoxyisoflavone (iristectrorigenin) (**33**) as the major phytochemicals of the polar fraction, with an occurrence of 25.89%, 21.95%, 20.97%, and 9.23%, respectively. The other five compounds that were identified included dodecanoic acid, methyl ester (0.37%) (**34**), 9,12-octadecadienoic acid (Z, Z)-, methyl ester (1.92%) (29), 9-octadecenoic acid (Z) (2.78%) (**35**), phytol (18.62%) (**26**), and hexadecanoic acid (16.89%) (**28**). Khalid et al. [66] first reported the isolation and characterization of the quinonemethide triterpene, (20α)-3-hydroxy-2-oxo-24-nor-friedela-1(10),3,5,7-tetraen-carboxylic acid-(29)-methylester (pristimerin) (**24**) from chloroform extract of the root bark of *M. senegalensis*. Zangueu et al. [22] chemically characterized the aqueous extract of *M. senegalensis* stem bark by the high-performance liquid chromatography-electrospray ionization-mass spectrometry (HPLC-ESI-MSn) and the study resulted in tentative identification of at least 36 secondary metabolites. Eight quercetin derivatives were found in the *M. senegalensis* extracts and were identified by comparison with an analytical reference. Compounds (**36**) and (**37**) were characterized as quercetin-O-dirhamnoside isomers. The other derivatives that were identified included quercetin-O-pentoside (**38**), quercetin-O-acetylhexoside (**39**), and quercetin-O-rhamnoside (**40**). Rutin (**41**) was identified by comparison with a reference standard. Compound (**42**) was only identified as a kaempferol derivative out of the six kaempferol derivatives that were detected. The other five derivatives were tentatively characterized as kaempferol-O-rutinoside (**43**), kaempferol-O-acetylhexoside (**44**), kaempferol-O-rhamnoside (**45**), kaempferol-O-rhamnosylpentoside (**46**), and kaempferol-O-di-rhamnoside (**47**). Two oxylipins were detected in the samples and characterized as oxo-dihydroxy-octadecenoic acids (**48**) and trihydroxy-octadecenoic acid (**49**). Epicatechin (**12**), which was previously shown from earlier studies conducted on the root bark of *M. senegalensis*, was detected [16] as well as another flavonoid, catechin (**50**). naringenin-6,8-di-C-hexoside (**51**) was tentatively characterized from the aqueous stems bark extract of *M. senegalensis*. Myricetin-O-rhamnoside (**52**) and phloretin-di-chexoside (**53**) were characterized and identified as well. Because of inconclusive data, a secondary metabolite that resembled ferulic acid could only be classified as a ferulic acid derivative (**54**). Proanthocyanidins dimers and trimers, all of them B-type, were characterized in the extracts and these included (epi) gallocathechin-(epi) catechin-(epi) catechin (**55**) as a trimer, (epi) catechin-(epi) catechin (**56**) procyanidin dimers, (epi)catechin-(epi)gallocathechin (**57**), and procyanidin trimers (epi) catechin-(epi) catechin-(epi) catechin (**58**). Two acids, citric acid (**59**) and vanillic acid derivative (**60**) were tentatively characterized. Channi and Biradar [25] identified secondary metabolites from the whole plant extract of *M. senegalensis* by GC–MS including hexadecanoic acid (**27**), 9-octadecenoic acid (Z) (**35**), 12-hydroxy-9-octadecenoic acid (**61**), methyl-1-cyclopentene-1-carbxylate (**62**), 12-hydroxy-9-octadecenoic acid, methyl ester (**28**), and 1, 1-dimethoxyacetone and 13 hexyloxacyclotridec-10-en-2-on (**63**). In another study, the main secondary metabolites isolated or identified from *Gymnosporia senegalensis* var spinosa methanol roots extracts were β-sitosterol (**22**), along with L-stachydrine (**64**) and two coumarins: scopoletin (**65**) and prenyletin (7-(3′-methyl-2′-butenyloxy)-6-methoxycoumarin) (**68**) [67]. Jain et al. [68] structurally characterized bioactive compounds from *G. senegalensis* petroleum-ether leaf extract using advanced analytical techniques like Fourier-transform infra-red (FTIR), GC-MS, and Nuclear Magnetic Resonance (1H-NMR) spectroscopy. Isolated compounds from petroleum-ether leaf extract included β-carotene (**67**), tetratetracontane (**68**), amyrin (**21**) and terpineol (**69**). Furthermore, GC-MS analysis of petroleum-ether leaf extract resulted in tentative identification of 17-octadecynoic acid (**70**), 1-iodo-2-methylundecane (**71**), disulfide, di-tert-dodecyl (**72**), dibutyl phthalate (**73**), α-D-glucopyranosiduronic acid,3-(5-ethylhexahydro-2,4,6-trioxo-5 pyrimidinyl)-1,1-dimethylpropyl 2,3,4-tris-0-(trimethylsilyl)-, methyl ester (**74**), hexadecaoic acid (**75**), picrotoxinin (**76**), curcumenol (**77**), carvacrol, TBDMS derivative (**78**), psi.,.psi.-carotene (**79**), astaxanthin (**80**), methyl glycocholate, 3 TMS derivative (**81**), pentadecane (**82**), tetratetracontane (**68**), β-amyrin (**21**), α-amyrin (**83**), olean-12-en-3-ol, acetate (3β) (**84**), 24-noroleana-3,12-diene (**85**), 24-norursa-3, 12-diene (**86**), α-terpineol (**69**), and L-α-terpineol (**87**). Eckelmann et al. [69] used MALDI-imaging-HRMS to visualize the occurrence as well as spatial and temporal distribution of maytansine (**88**) in an *M. senegalensis* plant during different seeding stages. The crude extracts of the seeds were analyzed by HPLC-HRMS and seed sections by MALDI-imaging-HRMS. Maytansine (**88**) was found to be distributed in the cotyledons and the endosperm with an increased accumulation towards the seed coat. The natural compound maytansine, a macrolide alkaloid with anti-tumor properties, was initially isolated from Maytenus serrata by Kupchan et al. [70]. Maytansine (**88**) is of high therapeutic interest because it is an anti-tumor drug with a unique structure. Okoye et al. [71] isolated six compounds from *M. senegalensis* methanol leaf extracts, including mayselignoside (**89**) and an unusual benzoyl malic acid derivative, benzoyl R-(+)-malic acid (**90**). Two known lignan derivatives (+)-lyoniresinol (**91**) and (−)-isolariciresinol (**92**), a known neolignan derivative dihydrodehydrodiconiferyl alcohol (**93**), and the triterpenoid, β-amyrin (**21**) were also isolated.

Tatsimo et al. [72] conducted a phytochemical investigation of the methanol leaf extracts of *Gymnosporia senegalensis* which led to the isolation of eighteen compounds using silica gel open-column chromatography. One new polyunsaturated fatty acid-derived monoglyceride, (2S)-1-O-(4′Z,7′Z,10′Z-octadecatrienoyl) glycerol (**94**), four galloylglucoside derivatives, including one new named (2R)-methyl [(6′-O-galloyl)-β-D-glucopyranosyloxy] phenylacetate (**95**), eleven phenolics, and two sterols, were reported in the study. Other isolated biomolecules included (S)-6′-O-galloylsambunigrin (**96**), (R)-6′-O-galloylprunasin (**97**), 1-O-β-D-(6′-O-galloyl)-glucopyranosyl-3-methoxy-5-hydroxybenzene (**98**), 3,5-dimethylgallate (**19**), quercetin (**99**), kaempferol 3-O-α-L-arabinofuranoside (**100**), quercetin 3-O-α-L-arabinofuranoside (**101**), kaempferol 3-O-α-L-rhamnopyranoside (**102**), quercetin 7-O-α-L-rhamnopyranoside (**103**), quercetin 3-O-β-D-xylopyranoside (**104**), quercetin-3-O-(6″-galloyl)-β-D-glucopyranoside (**105**), epicatechin 3-O-gallate (**106**), epigallocatechin-3-O-gallate (**107**), 1:1 isomeric mixture of hesperetin 3′-O-β-D-glucopyranoside (**108**), β-sitosterol (**22**), and β-sitosterol glucoside (**109**). Tatsimo et al. [73] isolated nine compounds from the leaves of *G. senegalensis* and the ethyl acetate fraction and non-polar fraction, which were rich in chlorophyll, including a new compound and a new megastigmane derivative. The new secondary metabolite was characterized as (3R*,5S*,6R*,7E,9ξ)-7-megastigmene-3,6,9-triol-3-O-β-D-(6′-O-galloyl)glucopyranoside (**110**), while other known compounds were 1-O-β-D-(6′-O-galloyl)-glucopyranosyl-3-methoxy-5-hydroxybenzene (**111**), 2,6-di-O-galloyl-β-D-glucose (112), phaeophytin A (**113**), phaeophorbide-a (**114**), Chlorine e6 trimethyl ester (**115**), (4Z,7Z,10Z)-octadecatrienoic acid (**116**), procyanidin B5 3,3′-di-O-gallate (**117**), and procyanidin B2 3,3′-di-O-gallate (**118**).

**Table 2:** Isolated and/or tentatively identified compounds from *Maytenus senegalensis* reported.

No	Secondary Metabolites	Plant Part	Detection/Isolation Method	References
**1**	(−)-4′-Methylepigallocatechin	Stem bark	Isolation, NMR	[65]
**2**	(−)-4″-Methylepigallocatechin 5-*O*-β-glucopyranoside	Stem bark	Isolation, NMR	[65]
**3**	(−)-Epigallocatechin	Stem bark, leaves	Isolation, NMR	[16,65]
**4**	(+)-4″-Methylgallocatechin 3”-*O*-β-glucopyranoside	Stem bark	Isolation, NMR	[65]
**5**	Epicatechin (4β→8) epigallocatechin	Stem bark	Isolation, NMR	[65]
**6**	(−)-Epicatechin (4β→4) (−)-4 0-methylepigallocatechin	Stem bark	Isolation, NMR	[65]
**7**	Epicatechin (4β→8) epicatechin (procyanidin B-2)	Stem bark	Isolation, NMR	[65]
**8**	Phloroglucinol 1-*O*-*β*-D-glucopyranoside	Stem bark	Isolation, NMR	[65]
**9**	3-Oxo-friedelan-20α-oic acid (maytenonic)	Roots, root bark	Isolation, NMR	[20,21]
**10**	Phenyldilactone, maysedilactone	Leaves	Isolation, NMR, HR-ESI-MS	[16]
**11**	9,10-Dihydroxy-4,7-megastigmadien-3-one	Leaves	Isolation, NMR	[16]
**12**	(−) Epicathechin	Leaves, stem bark	Isolation, HPLC-ESI-MS, NMR	[16,22]
**13**	(+) Gallocathechin	Leaves	Isolation, NMR	[16]
**14**	Procyanidin B-2	Leaves	Isolation, NMR	[16]
**15**	2,3-Dihydrokaempferol 3-O-β-D-glucopyranoside	Leaves	Isolation, NMR	[16]
**16**	Quercetin 3-O-β-D-glucopyranoside	Leaves	Isolation, NMR	[16]
**17**	Kaempferol 3-O-β-D-xylopyranoside	Leaves	Isolation, NMR	[16]
**18**	Quercetin 3-O-β-D-xylopyranoside	Leaves	Isolation, NMR	[16]
**19**	3,5-Dimethylgallate	Leaves	Isolation, NMR	[16,72]
**20**	Lupenone	Roots	Isolation, NMR, MS	[21]
**21**	β-Amyrin	Roots, leaves	Isolation, MS, GC-MS	[21,68,71]
**22**	β-Sitosterol	Roots, leaves	Isolation, NMR, MS	[21,67,72]
**23**	3-Hydroxy-20(29)-lupen-28-ol	Leaves	GC-MS	[23]
**24**	20α)-3-hydroxy-2-oxo-24-nor-friedela-1(10),3,5,7-tetraen-carboxylic acid-(29)-methylester (pristimerin)	Leaves, Root bark	GC-MS	[23,24,67]
**25**	2(4H)-Benzofuranone, 5,6,7,7a-tetrahydro-	Leaves	GC-MS	[23]
**26**	Phytol	Leaves, Root bark	GC-MS	[23,24]
**27**	*n*-Hexadecanoic acid	Leaves, root bark, whole plant	GC-MS	[23,24,25,26,27,28,29,30,31,32,33,34,35,36,37,38,39,40,41,42,43,44,45,46,47,48,49,50,51,52,53,54,55,56]
**28**	9,12-Octadecadienoic acid, methyl ester	Leaves, root bark, whole plant	GC-MS	[23,24,25]
**29**	*cis*-Vaccenic acid	Leaves	GC-MS	[23]
**30**	6-Methyl-cyclodec-5-enol	Leaves	GC-MS	[23]
**31**	5,6,7,7a-Tetrahydro-2(4H)-benzofuranone (isomintlactone)	Root bark	GC–MS	[24]
**32**	2H,6H-pyrano[3,2-b] xanthen-6-one (jacareubin)	Root bark	GC–MS	[24]
**33**	5,7,3′-Trihydroxy-6,4′-dimethoxyisoflavone (iristectrorigenin)	Root bark	GC–MS	[24]
**34**	Dodecanoic acid, methyl ester	Root bark	GC–MS	[24]
**35**	9-Octadecenoic acid (Z)	Root bark, whole plant	GC–MS	[24,25]
**36**	*Cis*-Quercetin-*O*-dirhamnoside	Stem bark	HPLC-ESI-MS	[22]
**37**	*trans*-Quercetin-*O*-dirhamnoside	Stem bark	HPLC-ESI-MS	[22]
**38**	Quercetin-*O*-pentoside	Stem bark	HPLC-ESI-MS	[22]
**39**	Quercetin-*O*-acetylhexoside	Stem bark	HPLC-ESI-MS	[22]
**40**	Quercetin-*O*-rhamnoside	Stem bark	HPLC-ESI-MS	[22]
**41**	Rutin	Stem bark	HPLC-ESI-MS	[22]
**42**	Kaempferol derivative	Stem bark	HPLC-ESI-MS	[22]
**43**	Kaempferol-*O*-rutinoside	Stem bark	HPLC-ESI-MS	[22]
**44**	Kaempferol-*O*-acetylhexoside	Stem bark	HPLC-ESI-MS	[22]
**45**	Kaempferol-*O*-rhamnoside	Stem bark	HPLC-ESI-MS	[22]
**46**	Kaempferol-*O*-rhamnosylpentoside	Stem bark	HPLC-ESI-MS	[22]
**47**	Kaempferol-*O*-di-rhamnoside	Stem bark	HPLC-ESI-MS	[22]
**48**	Oxo-dihydroxy-octadecenoic acid	Stem bark	HPLC-ESI-MS	[22]
**49**	Trihydroxy-octadecenoic acid	Stem bark	HPLC-ESI-MS	[22]
**50**	Catechin	Stem bark	HPLC-ESI-MS	[22]
**51**	Naringenin-6,8-di-C-hexoside	Stem bark	HPLC-ESI-MS	[22]
**52**	Myricetin-*O*-rhamnoside	Stem bark	HPLC-ESI-MS	[22]
**53**	Phloretin-di-Chexoside	Stem bark	HPLC-ESI-MS	[22]
**54**	Ferulic acid derivative	Stem bark	HPLC-ESI-MS	[22]
**55**	(epi) gallocathechin-(epi) catechin-(epi) catechin	Stem bark	HPLC-ESI-MS	[22]
**56**	(epi) catechin-(epi) catechin	Stem bark	HPLC-ESI-MS	[22]
**57**	(epi)catechin-(epi) gallocathechin	Stem bark	HPLC-ESI-MS	[22]
**58**	(epi) catechin-(epi) catechin-(epi) catechin	Stem bark	HPLC-ESI-MS	[22]
**59**	Citric acid	Stem bark	HPLC-ESI-MS	[22]
**60**	Vanillic acid derivative	Stem bark	HPLC-ESI-MS	[22]
**61**	12-Hydroxy-9- octadecenoic acid	Whole plant	GC–MS	[25]
**62**	Methyl-1-cyclopentene-1-carbxylate	Whole plant	GC–MS	[25]
**63**	1, 1-Dimethoxyacetone and 13 hexyloxacyclotridec-10-en-2-on	Whole plant	GC–MS	[25]
**64**	L-Stachydrine	Roots	Isolation, MP, IR, NMR	[67]
**65**	Scopoletin	Roots	Isolation, MP, IR, NMR	[67]
**66**	Prenyletin (7-(3′-methyl-2′-butenyloxy)-6-methoxycoumarin)	Roots	Isolation, MP, IR, NMR	[67]
**67**	β-Carotene	Leaves	Isolation, FTIR, NMR, GC-MS	[68]
**68**	Tetratetracontane	Leaves	Isolation, FTIR, NMR, GC-MS	[68]
**69**	Terpineol	Leaves	Isolation, FTIR, NMR, GC-MS	[68]
**70**	17-Octadecynoic acid	Leaves	GC-MS	[68]
**71**	1-Iodo-2-methylundecane	Leaves	GC-MS	[68]
**72**	Disulfide, di-tert-dodecyl	Leaves	GC-MS	[68]
**73**	Dibutyl phthalate	Leaves	GC-MS	[68]
**74**	α-D-Glucopyranosiduronic ac-id,3-(5-ethylhexahydro-2,4,6-trioxo-5 pyrimidinyl)-1,1-dimethylpropyl 2,3,4-tris-0-(trimethylsilyl)-, methyl ester	Leaves	GC-MS	[68]
**75**	Hexadecaoic acid	Leaves	GC-MS	[68]
**76**	Picrotoxinin	Leaves	GC-MS	[68]
**77**	Curcumenol	Leaves	GC-MS	[68]
**78**	Carvacrol, TBDMS derivative	Leaves	GC-MS	[68]
**79**	Psi.,.psi.-carotene	Leaves	GC-MS	[68]
**80**	Astaxanthin	Leaves	GC-MS	[68]
**81**	Methyl glycocholate, 3 TMS derivative	Leaves	GC-MS	[68]
**82**	Pentadecane	Leaves	GC-MS	[68]
**83**	α-Amyrin	Leaves	Isolation, GC-MS, FTIR, NMR	[68]
**84**	Olean-12-en-3-ol, acetate (3β)	Leaves	GC-MS	[68]
**85**	24-Noroleana-3,12-diene	Leaves	GC-MS	[68]
**86**	24-Norursa-3, 12-diene	Leaves	GC-MS	[68]
**87**	L-α-Terpineol	Leaves	GC-MS	[68]
**88**	Maytansine	Seeds	HPLC-HRMS	[69]
**89**	Mayselignoside	Leaves	Isolation. LC-ESI-MS, NMR	[71]
**90**	Benzoyl R-(+)-malic acid	Leaves	Isolation, LC-ESI-MS, NMR	[71]
**91**	(+)-Lyoniresinol	Leaves	Isolation, NMR	[71]
**92**	(−)-Isolariciresinol	Leaves	Isolation, NMR	[71]
**93**	Dihydrodehydrodiconiferyl	Leaves	Isolation, NMR	[71]
**94**	(2S)-1-O-(4′Z,7′Z,10′Z-Octadecatrienoyl) glycerol	Leaves	Isolation, HR-ESI-MS, APCI-MS, NMR, IR, UV	[72]
**95**	(2R)-methyl [(6′-O-Galloyl)-β-D-glucopyranosyloxy] phe-nylacetate	Leaves	Isolation, HR-ESI-MS, NMR, IR, UV	[72]
**96**	(S)-6′-O-Galloylsambunigrin	Leaves	Isolation, NMR	[72]
**97**	(R)-6′-O-Galloylprunasin	Leaves	Isolation, NMR	[72]
**98**	1-O-β-D-(6′-O-Galloyl)-glucopyranosyl-3-methoxy-5-hydroxybenzene	Leaves	Isolation, NMR	[72]
**99**	Quercetin	Leaves	Isolation, NMR	[72]
**100**	Kaempferol 3-O-α-L-arabinofuranoside	Leaves	Isolation, NMR	[72]
**101**	Quercetin 3-O-α-L-arabinofuranoside	Leaves	Isolation, NMR	[72]
**102**	Kaempferol 3-O-α-L-rhamnopyranoside	Leaves	Isolation, NMR	[72]
**103**	Quercetin 7-O-α-L-rhamnopyranoside	Leaves	Isolation, NMR	[72]
**104**	Quercetin 3-O-β-D-xylopyranoside	Leaves	Isolation, NMR	[72]
**105**	Quercetin-3-O-(6″-galloyl)-β-D-glucopyranoside	Leaves	Isolation, NMR	[72]
**106**	Epicatechin 3-O-gallate	Leaves	Isolation, NMR	[72]
**107**	Epigallocatechin-3-O-gallate	Leaves	Isolation, NMR	[72]
**108**	Hesperetin 3′-O-β-D-glucopyranoside Isomeric mixture	Leaves	Isolation, NMR	[72]
**109**	β-Sitosterol glucoside	Leaves	Isolation	[72]
**110**	(3R*,5S*,6R*,7E,9ξ)-7-Megastigmene-3,6,9-triol-3-O-β-D-(6′-O-galloyl)glucopyranoside	Leaves	Isolation, HR-ESI-MS, NMR	[73]
**111**	1-O-β-D-(6′-O-galloyl)-glucopyranosyl-3-methoxy-5-hydroxybenzene	Leaves	Isolation, NMR	[73]
**112**	2,6-Di-O-galloyl-β-D-glucose	Leaves	Isolation, HRMS-ESI, NMR	[73]
**113**	Phaeophytin A	Leaves	Isolation, NMR	[73]
**114**	Phaeophorbide-a	Leaves	Isolation, NMR	[73]
**115**	Chlorine e_6_ trimethyl ester	Leaves	Isolation, NMR	[73]
**116**	(4Z,7Z,10Z)-Octadecatrienoic acid	Leaves	Isolation, NMR	[73]
**117**	Procyanidin B5 3,3′-di-O-gallate	Leaves	Isolation, HRMS-ESI, NMR	[73]
**118**	Procyanidin B2 3,3′-di-O-gallate	Leaves	Isolation, NMR	[73]

Key: HR-ESI-MS; high-resolution electrospray ionization mass spectrometry. HPLC-HRMS; high-performance liquid chromatography-high resolution mass spectrometry. HPLC-ESI-MS; high-performance liquid chromatography-electrospray ionization-mass spectrometry. NMR; nuclear magnetic resonance. GC-MS; gas chromatography-mass spectrometry. MS; mass spectrometry. FTIR; Fourier-transform infra-red. HR-APCI-MS; atmospheric pressure chemical ionization and mass spectrometry. IR; infrared. UV; ultraviolet. MP; microwave plasma; MP.

### 3.5. Molecular Docking of the Secondary Metabolites

GC–MS analysis of the polar fraction of *G. senegalensis* root bark revealed the presence of several polar biomolecules, among which the polar secondary metabolites 5,6,7,7a-tetrahydro-2(4H)-benzofuranone (isomintlactone), ((20a)−3-hydroxy-2-oxo-24-nor-friedela-1(10),3,5,7-tetraen (pristimerin), and 2H,6H-pyrano[3,2-b] xanthen-6-one, (jacareubin) were identified as the major phytochemicals of the polar fraction, with percentage occurrences of 25.89%, 21.95%, and 20.97%, respectively. According to the molecular docking profile of prostaglandin E synthase, isomintlactone (**31**), pristimerin (**24**), and jacareubin (**32**) showed strong interactions with the crystal structure (Supplementary Material) of prostaglandin E synthase (PDB:4ALO) with binding affinities of −6.00 kcal/mol, −8.60 kcal/mol, and −8.30 kcal/mol, respectively. The ligand–receptor interaction analysis showed that the three compounds interacted with the prostaglandin E synthase through a variety of interactions, including Van der Waal forces, hydrogen bonds, and multiple π-interactions. Additionally, all the compounds established hydrophobic bonds with the prostaglandin E synthase binding pocket’s LEU69A and ARG73A residues [24].

### 3.6. Pharmacological Activities

Table 3 shows the reported studies on various pharmacological activities of twenty-one biomolecules isolated from *M. senegalensis*. Pharmacological activities such as antibacterial, antiplasmodial, anti-inflammatory antiproliferative, and cytotoxicity are indicated.

#### 3.6.1. Antimicrobial Activity

##### Antibacterial Activity

The antibacterial activity of the aqueous, hexane, and methanol extracts of *M. senegalensis*, and other 12 plant species traditionally used in Kenya, were tested using the agar diffusion method, in which the zones of inhibition around each disc were measured [49]. The ratio of the inhibition zone (mm) produced by the plant extract and the inhibition zone around the neomycin reference (mm) were used to express antibacterial activity. Both the stem bark and root bark extracts of *M. senegalensis* showed high activity, with the stem-bark methanolic extract showing the highest overall activity against *Staphylococcus aureus* at 1.68 ± 0.01. The stem-bark water extract exhibited strong activity as well, at 1.03 ± 0.27. Root bark extracts in methanol and water demonstrated antibacterial activity at 1.18 ± 0.17 and 0.7 ± 0.07, respectively. The antibacterial activity was assumed to be due to the presence of phenolic compounds that were previously identified from *Maytenus* species. After it was determined that methanolic preparations of the root bark of *M. senegalensis* exhibited antibacterial activity, Lindsey et al. [20] investigated the antibacterial activity of maytenonic acid that was isolated from the root bark. The antibacterial activity of maytenoic acid (9) against *Klebsiella pneumoniae*, *Escherichia coli*, and *Bacillus subtilus* was 98 µg/mL. Additionally, its MIC against *S. aureus* was just 195 µg/mL. A study to screen ten plant species collected from the Nelspruit Botanical Gardens was based on their uses by traditional healers in Limpopo Province of South Africa. *M. senegalensis* leaf samples extracted with hexane, dichloromethane (DCM), acetone, and methanolic were screened for antibacterial activity against *S. aureus* ATCC 29213, *Pseudomonas aeruginosa* ATCC 27853, *Enterococcus faecalis* ATCC 29212, and *E. coli* ATCC 25922 [74. All the leaf extracts (DCM, acetone, and methanol) of *M. senegalensis* were active against *E. faecalis* with MIC values of 0.02 mg/mL and an MIC of 0.06 mg/mL against the hexane extract. *M. senegalensis* root bark was tested for its antibacterial activity against *E. coli*, *Salmonella typhi*, and *S. aureus* at 150 mg/mL [74]. The methanol extract had the strongest impact on *S. aureus* (8 mm), while *E. coli* and *S. typhi* showed similar sensitivity (6 mm) to this extract. The anti-diarrhea properties of ethanol leaf extract from *M. senengalensis* were studied using rats [63]. The findings demonstrated that the percentage of protection following oral administration of varying extract dosages was dose-dependent and statistically significant (*p* ˂ 0.05) in comparison to the control. The rats had dose-dependent protection from the ethanolic extract, with 53.4% protection using 800 mg/kg. However, the group treated with 200 mg/kg and 400 mg/kg concentrations of the extract showed percentage protection of 13.9% and 32.4%, respectively, whereas the usual medication (loperamide 5 mg/kg) provided the maximum protection of 97.6%. Tatsimo et al. [72] determined the antibacterial activities of eighteen compounds from methanol leaf extracts of *G. senegalensis* against *S. aureus* NBRC 13276, *Bacillus subtilis* NBRC 3134, and *E. coli* NBRC 3972. All the tested biomolecules (compounds **19**, **22**, **94**–**109**) showed no growth inhibition at 50 µM against the tested bacteria. Tatsimo et al. [73] also evaluated the antibacterial activity of *G. senegalensis* methanol, hexane, ethanol, and butanol extracts and the same isolated compounds against *S. aureus* ATCC 25923, *P. aeruginosa* ATCC 27853, *E. coli* S2(1), and *Shigella flexneri* (SDINT). The ethyl acetate extracts displayed good antibacterial activity against *S. aureus* and *P. aeruginosa*, with MIC and minimum microbicidal concentration (MMC) values of 64 µg/mL against both bacteria, and moderate antibacterial activity with MIC and MMC values of 128 µg/mL against *E. coli* and *S. flexneri*. The methanol and n-butanol extracts displayed moderate antibacterial activities against all the tested bacteria with MICs between 128 µg/mL and 256 µg/mL. The *n*-hexane extract demonstrated poor activity with MICs between 1024 µg/mL and 2048 µg/mL. Based on the antimicrobial threshold of isolated compounds **110**, **113**, **114**, **116**, **117**, and **118**, moderate antibacterial activity was observed against all the tested bacteria with MIC values ranging from 16 to 128 µg/mL, whereas other compounds had poor antibacterial activity.

##### Antimycobacterial Activity

The antimycobacterial activity of *M. senegalensis* acetone and aqueous leaf extracts was tested against the H37Rv strain of *Mycobacterium tuberculosis* and drug-resistant *M. tuberculosis* strain (CCKO28469V) using an agar plate method. The acetone leaf extract had an MIC of 0.5 mg/mL while the water extract was not active against *M. tuberculosis* H37Rv strain. The extracts were further tested against the drug-resistant *M. tuberculosis* strain (CCKO28469V), which inhibited its growth at a concentration of 1.0 mg/mL [60]. A study by Mmushi et al. [75] examined the antimycobacterial activity of *M. senegalensis* extracts in hexane, DCM, acetone, and methanol against *Mycobacterium smegmatis*. The minimum inhibitory concentration (MIC) values for all the tested extracts ranged from >2.5 to 0.523 mg/mL, and acetone extracts had the lowest MIC values, at 0.523 mg/mL. The antimycobacterial activity of *Gymnosporia senegalensis* DCM leaf extract collected from the Limpopo Province in South Africa and its six fractions were assessed against the H37Rv strain of *M. tuberculosis* using a fluorescent microplate test [76]. The DCM extracts and its six fractions inhibited the growth of the *M. tuberculosis* H37Rv strain, with MIC_90_ and MIC_99_ values ranging from 21 to 54 μg/mL and from 27 to 86 μg/mL, respectively. The following sequence represents the order of strength for the tested samples’ overall antimycobacterial activity: DCM crude > SP2-1F5 > SP2-1F2 > SP2-1F6 > SP2-1F1 > SP2-1F3 > SP2-1F4 with MIC_90_ concentrations ranging from 21.4 to 54.2 μg/mL.

##### Antifungal Activity

The methanol, hexane, ethanol, and butanol leaf extracts of *G. senegalensis* and bioactive molecules were tested for their antifungal activity against three yeast strains, including *Candida albicans* ATCC 10231, *C. tropicalis* PK233, and *Cryptococcus neoformans* H99 [73]. The ethyl acetate and butanol extracts showed moderate antifungal activity against all the tested fungi with MIC values of 128 µg/mL and 256 µg/mL, respectively. The methanol and hexane extracts had poor antifungal activities with MIC values ranging from 512 µg/mL to 2048 µg/mL. All the compounds had moderate to poor activities with MIC values from 16 µg/mL to >256 µg/mL. Compounds **113**, **114**, **115**, **117**, and **118** had MIC values from 16 µg/mL to 64 µg/mL. The new compound (**110**) had poor antifungal activity against all the fungi tested, with MIC values ranging from 64 µg/mL to >256 µg/mL.

#### 3.6.2. Antiproliferative Activity

Khalid et al. [66] determined the cytotoxicity of quinonemethide triterpene, (20α)-3-hydroxy-2-oxo-24-nor-friedela-1(10),3,5,7-tetraen-carboxylic acid-(29)-methylester (pristimerin) (**24**), isolated from *M. senegalensis* root bark, extracted with chloroform. This was undertaken by measuring the inhibition of the human peripheral blood lymphocyte ability of perforation when stimulated by the mitogen phytohemagglutinin (PHA) and showed an IC_50_ of 6.8 ± 0.8 μg/mL. The in vitro cytotoxicity activity of *M. senegalensis* ethanol root bark, which was obtained from Tanzania, was evaluated against rat skeletal myoblast (L-6) cells [77]. Additionally, the selectivity indices (SI) were also calculated. The extract exhibited an SI of over 10,000 and an IC_50_ of over 90 μg/mL on Rat L-6 cells. Eleven isolated compounds from the leaves of *M. senegalensis* 7-(1,2-dihydroxyethyl)-4a-hydroxy-4-(4-hydroxyphenyl)tetrahydro-2H-furo[3,4-b]pyran-2,5 (3H)-dione (maysedilactone) (**10**), 9,10-dihydroxy-4,7-megastigmadien-3-one (**11**), (−) epicathechin (**12**), (+) gallocathechin (**13**), (−) epigallocathechin (**3**), procyanidin B-2 (**14**), 2,3-dihydrokaempferol 3-O-β-D-glucopyranoside (**15**), quercetin 3-O-β-D-glucopyranoside (**16**), kaempferol 3-O-β-D-xylopyranoside (**17**), quercetin 3-O-β-D-xylopyranoside (**18**), and 3,5-dimethylgallate (**19**) were tested for cytotoxicity against a mouse lymphoma cell line (L5178Y) [16]. Only (−) epigallocathechin (**3**) showed a high cytotoxicity and completely inhibited cell growth at the investigated dose of 10 µg/mL. The following compounds, including (+) gallocathechin (**13**), quercetin 3-O-β-D-glucopyranoside (**16**), kaempferol 3-O-β-D-xylopyranoside (17), and quercetin 3-O-β-D-xylopyranoside (**18**) showed very mild cytotoxic activity while the other compounds were inactive at the same dose. Unfortunately, the new compound, maysedilactone, did not show any cytotoxic activity at the tested dose of 10 µg/mL [16]. Makgatho et al. [76] studied the cytotoxicity of *M. senegalensis* fractions against RAW 264.7 murine macrophage cells. The SP2-1F3 fraction had notable activity at concentrations of 62 μg/mL and above, while portions SP2-1F1 and SP2-1F5 suppressed cell growth at doses ranging from 125 to 500 μg/mL. Bah et al. [77] evaluated the in vitro toxicity of *M. senegalensis* methanolic root extract using the MTT (3-(4, 5-dimethylthiazol-2-yl)-2, 5-diphenyl tetrazolium bromide), neutral red, and LDH (Lactate Dehydrogenase) activity assays on human epithelial (Caco-2) cells and human liver cancer (HepG2) cells. Apoptotic activity was evaluated by electrophoretic migration of the DNA extract to determine a potential fragmentation. Results showed that the methanol root extract had some cytotoxicity on all the cell types used in the study. On an MTT assay, the crude extract had an IC_50_ of 29.07 μg/mL and 39.40 μg/mL for Caco2 and HepG2, respectively. The extract had an impact on cells as measured by the neutral red test at 80 μg/mL, with the IC_50_ values of 60.95 μg/mL on Caco2 and 75.24 μg/mL on HepG2 cells. Overall, cell viability decreased as concentrations increased up to 80 μg/mL. The Caco2 and HepG2 cells treated with extract for 48 h had comparable LDH activity levels, with toxicity of at least 10 μg/mL, and IC_50_ values of 25.38 μg/mL and 24.40 μg/mL, respectively. In both the Caco-2 and HepG2 cell lines treated with 10 μg/mL and 100 μg/mL methanolic extracts, respectively, DNA fragments the size of 200 kb had increments of 200 bp. Tatsimo et al. [72] reported the antiproliferative activity of eighteen isolated compounds from *G. senegalensis* methanol leaf extracts against the colon cancer cells (DLD1), breast cancer cells (MCF7), and gastric cancer cells (MKN45). Compounds **92**, **99**, **101**, **22**, and **109** at 50 µM displayed a relatively weak to moderate decrease in viability against DLD1, MCF7, and MKN45 cancer cell lines. Compound **108** at 50 μM showed a relatively strong decrease of viability to 69.6% against DLD1 colon cancer cells, 65.6% against MCF7 breast cancer cells, and 55.7% against MKN45 gastric cancer cells. Compound **109** had the strongest decrease of cell viability by 59.0% against MKN45 cells at 50 μM, while the cell viability against DLD1 and MCF-7 cells was more than 70%. Compound **102** had 68.4% cell viability against MCF-7 cells at 50 μM. Compounds **94** and **100** had a decrease of cell viability between 67.0 and 68.0% against MKN45 cells, respectively. Compounds **95**, **96**, **99**, **98**, **19**, **100**, **102**, **103**, **104**, **105**, **106**, **107**, and **108** had cell viability >70% against MKN45 cells. Nabende [78] investigated the in vitro antiproliferative activity of *G. senegalensis* methanol and aqueous bark and leaf extracts against a mouse mammary breast cancer cell line (4T1 ATCC^®^ CRL-2539TM), a mouse colon cancer cell line (CT26.WT-ATCC^®^ CRL-2638TM), and Vero cells (monkey kidney cells). Methanol extracts of *M. senegalensis* stem bark had the lowest IC_50_ value of 32.96 ± 2.91 µg/mL against the mouse mammary breast cancer cells. *M. senegalensis* aqueous leaf, methanol leaf, and aqueous stem extracts had IC_50_ values of >1000 µg/mL, 256.41 ± 4.77 µg/mL, and >1000 µg/mL against the mouse mammary breast cancer cells. The methanol stem bark and methanol leaf extracts of *M. senegalensis* had IC_50_ values of 2.32 ± 0.17 μg/mL and 4.18 ± 0.14 μg/mL, respectively, against the colon cancer cells. *M. senegalensis* aqueous leaf, methanol leaf, aqueous stem bark, and methanol stem bark extracts had IC_50_ values of >1000 µg/mL, 464.04 ± 0.02 µg/mL, >1000 µg/mL, and 74.59 ± 3.21 µg/mL, respectively, against the Vero cells.

#### 3.6.3. Anti-Inflammatory Activity

The cyclooxygenase (COX-1) assay was used to evaluate the anti-inflammatory properties of *M. senegalensis* root bark, stem bark, and leaves extracted with hexane, methanol, and water at a test concentration of 500 µg/mL [49]. Methanol root bark (93.0 ± 1.0), aqueous root bark (84.0 ± 2.5), and methanol stem bark (81.0 ± 1.0) extracts exhibited the highest COX-1 percentage inhibition, with activity exceeding the 70% inhibition threshold, which is regarded as significant. Sosa et al. [21] evaluated the topical anti-inflammatory effects of *M. senegalensis* root extracts by assessing the reduction in mice’s ear oedema caused by Croton oil. With a potency comparable to that of the NSAID reference medication indomethacin (ID_50_ = 84 µg/cm2 and 93 µg/cm^2^, respectively), the chloroform extract exhibited the greatest anti-inflammatory effect, significantly reducing the oedematous response. Maytenoic acid (9) was isolated by fractionating the hexane and chloroform extracts. It had a dose-dependent antiphlogistic effect (ID_50_ = 0.11 µmol/cm^2^) that was only three times lower than that of hydrocortisone (ID_50_ = 0.04 µmol/cm^2^) and twice that of indomethacin (ID_50_ = 0.26 µmol/cm^2^). The topical anti-inflammatory efficacy of *M. senegalensis* methanol and chloroform root extracts were evaluated at 300 µg/cm^2^. The oedematous reaction was significantly reduced by each extract, ranging from 20% for the methanol extract to 92% for the chloroform extract. When the chloroform extract was examined further, it was found to have a potency similar to indomethacin, although five times less active than hydrocortisone. The extract also demonstrated a dose-dependent reduction in the production of oedema. It had an ID_50_ value of 84 µg/cm^2^, which was somewhat less than that of indomethacin (ID_50_ = 93 µg/cm^2^), but hydrocortisone had an ID_50_ of 16 µg/cm^2^ [21]. Maytenoic acid (**9**), lupenone (**20**), and β-amyrin (**21**) isolated from hexane and chloroform extracts were evaluated for their anti-inflammatory activity in comparison to indomethacin and hydrocortisone. Maytenoic acid (**9**) demonstrated a significant dose-dependent oedema inhibition, reducing the oedematous response by 29% at a dose of 0.03 µmol/cm^2^, and by over 90% at the highest dose (1 µmol/cm^2^). Lupenone (**20**) caused a 26% decrease in oedema at 0.1 µmol/cm^2^, and at the maximum dose (1 µmol/cm^2^), it achieved a 65% reduction. This is comparable to β-amyrin (**21**), which caused oedema inhibitions ranging from 19% to 62% at the same dose levels. Maytenoic acid (**9**), with an ID_50_ of 0.11 mmol/cm^2^, was twice as active as indomethacin (ID_50_ = 0.26 µmol/cm^2^), but three times less effective than hydrocortisone (ID_50_ = 0.04 µmol/cm^2^). Additionally, its potency was four times that of lupenone (ID_50_ = 0.49 µmol/cm^2^) and five times that of β-amyrin (ID_50_ = 0.57 µmol/cm^2^) [21]. In another study, rat’s paw oedema, induced by carrageenan, was treated with 70% ethanol extracts of *M. senegalensis*. After 6 h, rats who received an intraplanar injection of carrageenan showed a statistically significant increase in paw volume (162.6% + 17.5) in comparison to the control group. Leaf extracts from *M. senegalensis* reduced paw oedema by 33%, which was comparable to indomethacin. The impact of oral *M. senegalensis* leaf and stem extracts on the development of rat paw oedema caused by carrageenan was also evaluated [13]. This study was conducted to investigate the analgesic effects of the leaf methanolic extract of *M. senegalensis* using two models: acetic acid-induced writhing in mice and formalin-induced pain in rats [79]. The extract significantly (*p* < 0.001) decreased the number of acetic acid-induced abdominal constrictions in a dose-dependent manner at 150 and 300 mg/kg. The typical non-steroidal analgesic and anti-inflammatory medication, Piroxicam, had a lower inhibition of abdominal constriction (*p* < 0.001) than the 300 mg/kg dose. Makgatho et al. [76] evaluated the in vitro anti-inflammatory properties of a crude dichloromethane leaf extract of *M. senegalensis* and its six fractions by determining the production of tumor necrosis factor-alpha (TNF-α) in RAW264.7 murine macrophage cells. From 125 to 500 μg/mL, fractions SP2-1F1 and SP2-1F5 exhibited notable activity, whereas fraction SP2-1F3 showed a decrease in nitric oxide production at concentrations ranging from 62 to 500 μg/mL. TNF-α production by RAW cells was also suppressed by all three fractions (SP2-1F1, SP2-1F3, and SP2-1F5) between 125 and 500 μg/mL. The anti-inflammatory and antinociceptive properties of the methanol root extract of *M. senegalensis* were assessed by Umar et al. [28]. The effect of the hydroalcoholic extract of *M. senegalensis* roots on the bronchial hyperleukocytosis that happens during airway inflammation was assessed in this study. Leukocytes in general, and eosinophils in particular, were significantly blocked from infiltrating the bronchi by the extract (*p* < 0.001). In mice’s bronchoalveolar lavage fluid (BALF), ovalbumin (OVA) sensitization caused a notable increase in the number of leukocytes overall and the proportion of eosinophils in comparison to the control. When BALF was treated with the extract and cetirizine, the number of these leukocytes was significantly reduced. A larger reduction in activity was observed at the dose of 500 mg/kg [62]. The acetic acid-induced pain model and egg albumin-induced paw oedema were used to test the anti-inflammatory and analgesic properties of *M. senegalensis* leaf extracts. At 75 mg/kg and 150 mg/kg bw, the purified fraction had an analgesic efficacy of 43.35 ± 4.98% and an anti-inflammatory effect of 53.16 ± 4.09 and 60.76 ± 7.54, respectively [23]. Paw oedema caused by egg albumin was reduced in a dose-dependent manner by the crude extract at 400 and 800 mg/kg bw. In comparison to the reference standard, acetylsalicylic acid, which showed inhibition of 70.96%, the extract showed 51.92% and 54.66% inhibition of paw oedema. The extract showed the most significant (*p* < 0.05) nociceptive suppression of thermal stimulation at the higher dose. Kpoyizoun et al. [19] evaluated the anti-inflammatory activity of the *M. senegalensis* roots hydroethanolic extract and its fraction. The number of inflammatory cells in BALF with OVA sensitization was significantly increased. However, treatment with the extract and its fraction reduced this number (*p* < 0.01; *p* < 0.001). The hydroethanolic extract 500 mg/kg (50.68%) and supernatant fraction 250 mg/kg (62.10%) demonstrated a substantial drop in total leukocytes (*p* < 0.01; *p* < 0.001), while all treated groups showed a significant decrease in eosinophil and neutrophil counts (*p* < 0.05; *p* < 0.01; *p* < 0.001). Comparatively, the supernatant fraction of 250 mg/kg induced the best reduction of cell number in BALF (91.61% eosinophils and 71.47% neutrophils). *M. senegalensis* extract and its fractions significantly reduced the number of inflammatory cells in BALF, lowered the quantity of MDA, and increased the level of glutathione in lung homogenate (*p* < 0.05; *p* < 0.01; *p* < 0.001). The extract and its fraction of anti-inflammatory activities were validated by histological studies. The antiedematogenic (paw oedema caused by egg albumin) and antinociceptive (hot plate) properties of the crude methanol extract and the polar fraction from *M. senegalensis* root bark were investigated [24]. At 400 and 800 mg/kg, the crude methanol extracts inhibited paw oedema by 51.92 ± 2.34 and 54.66 ± 1.35%, while at 200 and 300 mg/kg, the polar fraction inhibited paw oedema by 57.12 ± 3.35 and 58.22 ± 2.90%. Furthermore, a dose-dependent antinociceptive efficacy (*p* < 0.01) against heat-induced pain in rats was shown using crude methanol extract. According to Ndako et al. [24], the polar fraction and crude methanol extract demonstrated antiedematogenic and antinociceptive properties. The polar fraction and crude methanol extract effectively (*p* < 0.01) inhibited the dose-independent rat paw oedema caused by egg albumin. The polar fraction induced 57.12 ± 3.35 and 58.22 ± 2.90% suppression of paw oedema at 200 and 300 mg/kg, respectively, whereas the crude methanol extract caused 51.92 ± 2.34 and 54.66 ± 1.35 percentage inhibition of paw oedema at 400 and 800 mg/kg. Furthermore, the crude methanol extract showed a dose-dependent antinociceptive efficacy (*p* < 0.01) against rats’ thermally generated discomfort. However, the polar fraction exhibited no significant (*p* > 0.05) antinociceptive effect at 200 and 300 mg/kg in rats [24].

#### 3.6.4. Antiparasitic Activity

Gessler et al. [29] determined the antimalarial efficacy of *M. senegalensis* stem and root bark collected from Tanzania’s Morogoro region against the chloroquine-sensitive NF54 strain and the multidrug-resistant *P. falciparum* strain K1. The ethyl acetate fraction of *M. senegalensis* stem bark exhibited the strongest antimalarial activity, with an IC_50_ value of 0.16 µg/mL. Both root bark and stem bark crude ethanol and petroleum ether fractions had IC_50_ values of less than 10 µg/mL against *P. falciparum* strain K1 in vitro. The aqueous fractions of stem bark and root bark had IC50 values of only 80 µg/mL and 85 µg/mL, respectively. The antiplasmodial efficacy of *M. senegalensis* stem bark and leaf extracts was evaluated against the *P. falciparum* chloroquine-sensitive strain 3D7, and the chloroquine-resistant strain Dd2 [9]. The IC_50_ values for the stem bark and leaves of *M. senegalensis* were as follows: 3.9 µg/mL and 5.1 µg/mL for the *P. falciparum* 3D7 strain, and 10 µg/mL and 65 µg/mL for the *P. falciparum* Dd2 strain. Column chromatography was used to purify the dichloromethane extract, and the high non-polar fraction had the strongest antiplasmodial activity, with fraction 2 exhibiting an IC_50_ value of 0.5 µg/mL. Extracts of 23 plant species, including *M. senegalensis*, used popularly against schistosomiasis in Zimbabwe were screened for their anthelmintic effect [80]. In vitro studies were conducted on the trematode *Schistosoma mansoni*’s schistosomules and the cestode *Hymenolepis diminuta*’s cysticercoids. Aqueous leaf and stem extracts of *M. senegalensis* were effective against *Hymenolepis diminuta* cestodes at concentrations of 25 mg/mL after one hour and 6.3 mg/mL after 24 h. Using the roots and root bark, similar patterns were seen, with the extracts showing efficacy after 1 h and 24 h at doses of 25 mg/mL and 2.5 mg/mL after an hour, and 5 mg/mL and 2.5 mg/mL after 24 h, respectively. At a dosage of 100 mg/mL, aqueous root bark extracts of *M. senegalensis* showed some action on *Schistosoma mansoni* schistosomules. Clarkson et al. [81] tested *M. senegalensis* extracts for in vitro activity against *P. falciparum* strain D10 using the parasite lactate dehydrogenase assay. In vitro antiplasmodial activity of the plant extracts against *P. falciparum* D10 was observed for *M. senegalensis* dichloromethane (DCM) (IC_50_ = 15.5 µg/mL) and water (IC_50_ >100 µg/mL) root extracts, and DCM (IC_50_ = 42 µg/mL), DCM: MeOH (1:1) (IC_50_ = 48.3 µg/mL) and water (IC_50_ > 100 µg/mL) stem extracts. In vivo antimalarial efficacy against chloroquine (CQ)-tolerant *Plasmodium berghei* NK65 in mice was assessed using methanolic leaf and root bark extracts from *M. senegalensis*, either by themselves or in combination with CQ [82]. Khalid et al. [66] determined the in vitro antiplasmodial activity of the isolated compound, pristimerin (**32**), from the root bark of *M. senegalensis* against a chloroquine-resistant strain (Dd2) of *P. falciparum*. Pristimerin (**32**) had an IC_50_ of 0.5 μg/mL, and the in vitro antileishmanial activity performed on promastigotes of Leishmania major was IC_50_ = 6.8 ± 0.8 μg/mL. The second fraction of the root bark of *M. senegalensis* had the highest antimalarial activity with an IC_50_ of 1.6 μg/mL and antileishmanial activity with an IC_50_ of 6.3 μg/mL activities. Methanolic leaf extracts of *M. senegalensis* displayed no parasitaemia suppression on day 4 post-infection for mice but induced significant suppression of 55.4% on day 11 post-infection using a combination of CQ and methanolic leaf extracts of *M. senegalensis*. On day 4 post-infection, mice treated with methanolic root bark extracts of *M. senegalensis* showed parasitaemia suppression of only 2.4%, but on day 11 post-infection, mice treated with a combination of CQ and methanolic root bark extracts of *M. senegalensis* showed a considerable suppression of 56.2%. On the ninth day after infection, mice that were given methanolic root bark and leaf extracts of *M. senegalensis* had a 20% survival rate. On day 14 following infection, however, mice’s survival rates for methanolic leaf extracts were 40%, while the survival rates for root bark extracts stayed constant. The anti-plasmodial potential of twelve plant species, including *M. senegalensis*, traditionally used in preparing herbal remedies for malaria in the Kilifi and Tharaka districts of Kenya, was evaluated. The extracts were tested against chloroquine-sensitive (NF54) and resistant (ENT30) *P. falciparum* strains in vitro using a 3 Hypoxanthine assay [83]. The root-bark methanol extract was efficacious against *P. falciparum* NF54 with IC_50_ values of 38.24 ± 2.86 µg/mL and against *P. falciparum* ENT30 with IC_50_ values of 42.78 ± 4.12 µg/mL. For *P. falciparum* NF54, the root-bark water extract exhibited moderate activity, with IC_50_ values of 93.38 ± 4.35 µg/mL, and for *P. falciparum* ENT30, 96.56 ± 6.54 µg/mL. Malebo et al. [84] tested *M. senegalensis* ethanol root bark from Tanzania for antiplasmodial, anti-trypanosomal, and antileishmanial activity as well as selectivity indices (SI). The extract had an IC_50_ of 2.05 ± 0.68 μg/mL and an SI of >43.9 against *P. falciparum* K1, an IC_50_ of 12.2 ±1.6 μg/mL and an SI of >7.4 against *Trypanosoma brucei* rhodesiense STIB 900, and an IC_50_ of 16.5 ± 2.32 μg/mL and an SI of >5.50 against axenic *Leishmania donovani* MHOM-ET-67/82. Muthaura et al. [85] carried out a study in Kenya to evaluate medicinal plants traditionally used for the management of malaria. The plant samples collected, including *M. senegalensis*, were tested for antiplasmodial activity against chloroquine-sensitive (D6) and resistant (W2) *P. falciparum* using the ability of extracts, prepared from the plant species, to inhibit the incorporation of [G-3H] hypoxanthine into the malaria parasites. Methanol root bark had an IC_50_ of 4.7 µg/mL against the *P. falciparum* chloroquine-sensitive D6 strain and an IC_50_ of 9.8 µg/mL against the *P. falciparum* resistant W2 strain. The aqueous extract had an IC_50_ of >100 against both parasites. Malebo et al. [32] investigated the in vivo antiplasmodial effects of *M. senegalensis* ethanol extracts in mice. The results of the in vivo antiplasmodial effect of the ethanol extract showed a significant dose-dependent decrease in malaria parasite counts in mice infected with *P. berghei* (*p* = 0.001). The *M. senegalensis* ethanol extract dosages ranging from 25 to 100 mg/kg body weight exhibited a considerable suppression of parasitaemia ranging from 88.5% to 98.1% (*p* = 0.001), according to smears taken on day 7 after infection. When the *M. senegalensis* ethanol extract was administered to mice at a dose of 100 mg/kg body weight, the greatest reduction in parasitaemia was seen with 98.1% suppression. The in vivo antiplasmodial activity of the extract was expressed by the dose inhibiting 50%, 75%, 90%, and 99% of parasite growth (ED50, ED75, ED90, ED99). The *M. senegalensis* ethanol extract showed strong in vivo antiplasmodial action in mice infected with *P. berghei*, as evidenced by the effective dose that cured 50% of the test animals (ED50), which was determined to be 3.3 mg/kg body weight. Moreover, the extract showed additional encouraging effective doses (ED75, ED90, and ED99) at determined dosages of 10.2 mg/kg, 28.4 mg/kg, and 166.5 mg/kg body weight, respectively. The anthelmintic activity of *M. senegalensis* acetone/water (70/30%) leaf extracts was assessed by Mengistu et al. [86]. Furthermore, their role in the inhibition of *Haemonchus contortus* larval exsheathment was also examined, and a relationship between inhibition of larval exsheathment was established. Larval exsheathment considerably suppressed (*p* < 0.001) the browsing plant extracts in a dose-dependent manner. After 60 min, the browsing extracts had a linear (*p* < 0.001) decrease in exsheathment as the dose increased. For every extract, the relationship between dosages and exsheathment was quadratic. The EC_50_ values, which were below the detection limit for *M. senegalensis*, also demonstrated the effect of browsing plant extracts on exsheathment. Overall, the phenolic and non-phenolic chemicals in the browsing plant extracts suppress larvae and show anthelmintic properties against *H. contortus*. Zangueu et al. [22] evaluated the anthelmintic activity of the aqueous stem bark extract of *M. senegalensis* using an egg hatch assay (EHA), larval migration inhibition assay (LMIA), and adult worms’ motility inhibition assay (AMIA). The aqueous extract concentrations examined in the egg hatch assay varied from 31.86% at 75 μg/mL to 54.92% at 2400 μg/mL 48 h after exposure to eggs, and they significantly (*p* < 0.01) inhibited egg hatching in a concentration-dependent manner. The aqueous extract of *M. senegalensis* significantly (*p* < 0.05) inhibited larval migration in a concentration-dependent manner according to the larval migration inhibition assay. At the maximum dose of 2400 μg/mL, the inhibition was 37.77%. The application of polyvinyl polypyrrolidone (PVPP) indicated that flavonoids and tannins were partially responsible for the effect, but other biochemical components were also implicated, as the larval migration was slowed by 15.5%. *M. senegalensis* was associated with reduced worm motility 24 h after exposure in the adult worm motility inhibition assay (*p* < 0.05) when compared to phosphate-buffered saline as a control. Sixty-six percent of the worms in the plant extract (2400 μg/mL) wells were either dead or immobilized at this stage. Umar et al. [28] determined the antiplasmodial activity of *M. senegalensis* methanol root extract on in vivo mice models. At 400 and 800 mg/kg bw, the extract caused 58.88% and 58.49% inhibition of parasitemia, using the *Plasmodium berghei* NK65 chloroquine-sensitive strain. The purified fraction of *M. senegalensis* leaf was evaluated against *Plasmodium chabaudi* and *Plasmodium berghei* using a 4-day suppressive test on Swiss albino mice [23]. At 75, 150, and 300 mg/kg bw, the purified fraction of *M. senegalensis* leaf exhibited dose-dependent antiplasmodial activity with percentage curative effects of 15.24 ± 0.89%, 45.70 ± 3.43%, and 48.50 ± 4.56% against *P. chabaudi* and 44.25 ± 3.21%, 72.74 ± 6.54%, and 76.30 ± 8.32% against *P. berghei*. Domo et al. [87] assessed certain liver enzymes in albino rats that had been infected with Schistosome cercariae and given methanol and water extracts of *M. senegalensis*. The extracts were well tolerated by the rats as therapeutic agents. For *M. senegalensis*, the total protein was 64.3–74.3 g/L for water extracts and 69.0–77.9 g/L for methanolic extracts. The alkaline phosphate values for *M. senegalensis* extracts ranged from 145 to 226 IU/L for methanol and from 143 to 234 IU/L for water. For *M. senegalensis*, the aspartate aminotransferase levels were 7.7–8.7 IU/L when using methanol extracts and 7.1–8.8 IU/L when using water extracts. For *M. senegalensis* in methanol, the alkaline aminotransferase value ranged from 5.1 to 6.3 IU/L, while for water extracts they ranged from 4.2 to 6.3 IU/L. For *M. senegalensis*, the total bilirubin levels in the methanol and water extracts were 6.3–18.9 μm/L and 4.2–6.3 μm/L, respectively. Ndako et al. [24] tested the crude methanol extract and the polar fraction from *M. senegalensis* root bark for antimalarial activity against *P. berghei* parasites in mice. In comparison to the crude methanol extract at 800 mg/kg (29.72 ± 0.23%; *p* < 0.05), the polar fraction at 200 and 300 mg/kg showed greater suppression of malaria infection (43.70 ± 1.16% and 47.69 ± 1.03% all *p* < 0.01) and extended the mice’s survival days, but by less than the chloroquine (73.13 ± 2.34% *p* < 0.001). The polar fraction and crude methanol extract substantially inhibited the *P. berghei* parasite’s ability to replicate in mice. The polar fraction at 200 mg/kg and 300 mg/kg had a higher therapeutic effect (43.70 ± 1.16% and 47.69 ± 1.03% all *p* < 0.01) against the parasite than did the crude methanol extract at 800 mg/kg (29.72 ± 0.23% *p* < 0.05), even though chloroquine showed the highest suppression of parasite replication and curative effect (73.13 ± 2.34% *p* < 0.001). Interestingly, both the crude methanol extract at 800 mg/kg as well as the polar fraction (200 mg/kg and 300 mg/kg) prevented the parasite-induced loss of packed cell volume (PCV) and prolonged the survival of the animals when compared with the control counterpart. Collectively, these findings strongly suggest that the polar fraction suppressed the replication of *P. berghei* infection and prolonged the survival days of the animal. The larvicidal efficacy of *M. senegalensis* leaf extracts was investigated for the biocontrol of *Culex quinquefasciatus* [25]. The larvicidal LC_50_ of *M. senegalensis* ethyl acetate, chloroform, and hexane extracts against *C. quinquefasciatus* were 87.87 ppm, 177.20 ppm, and 164.53 ppm, respectively. The ethyl acetate extract showed relatively high efficacy against *C. quinquefasciatus*, with an LC_50_ value of 87.87 ppm. However, the hexane and chloroform extract had low larvicidal activity against *C. quinquefasciatus*.

#### 3.6.5. Antioxidant Activity

Makgatho et al. [76] determined the antioxidant activity of the crude dichloromethane (DCM) leaf extract of *Gymnosporia senegalensis* (*M. senegalensis* synonym) and its six fractions using DPPH (2,2-diphenyl-1-picrylhydrazyl) and ferric ion reducing power. Significant (*p* < 0.05) antioxidant activity was demonstrated by the leaf extract fractions (SP2-1F1, SP2-1F3, and SP2-1F5) at doses ranging from 125 to 500 μg/mL. Additionally, the DCM crude extract demonstrated considerable ferric-reducing capacity at 250 and 500 μg/mL (*p* < 0.05). There was no antioxidant activity observed in the other tests (*p* > 0.05). The hydroalcoholic extract of the roots of *M. senegalensis* was tested to determine its in vitro antioxidant capacity using DPPH and in vivo antioxidant activity using the malondialdehyde (MDA) assay [62]. The in vitro and in vivo antioxidant tests revealed the reducing effect and the inhibitory membrane lipoperoxidation potential of the extract. The hydroethanolic extract of *M. senegalensis* showed an IC_50_ value of 120 μg/mL on DPPH which was lower than the IC_50_ value of the positive control used, ascorbic acid (170 μg/mL). The purpose of the MDA assay was to assess oxidative stress, specifically lipid peroxidation in the asthma model, and the impact of the *M. senegalensis* extract on the stress. Compared to the control group, the MDA concentration in sensitized mice increased significantly (*p* < 0.01). At dosages of 250 mg/kg (38.91%) and 500 mg/kg (55.15%), the extract significantly decreased this concentration (*p* < 0.05, *p* < 0.01). Jain et al. [64] reported the antioxidant potential of *Gymnosporia senegalensis* leaf, stem, and bark extracts using the 2,2-diphenyl-1-picrylhydrazyl (DPPH), hydrogen peroxide scavenging (H_2_O_2_), superoxide anion radical scavenging, metal chelating ferrous ion, ferric reducing antioxidant power (FRAP), and total antioxidant capacity (TAC) assays. The antioxidant analysis revealed that the IC_50_ and percentage (%) inhibition were dose dependent. The highest antioxidant activity was found in the methanol extract of the leaf for DPPH (40.9 ± 0.9 μg/mL), in the chloroform extract of the stem for H_2_O_2_ (88.8 ± 1.12 μg/mL), in the aqueous extract of the bark for superoxide anion radical scavenging activity (43.9 ± 0.15 μg/mL), for the metal chelating ferrous ion activity (26.9 ± 0.11 μg/mL), in the chloroform extract of the leaf for the metal chelating ferrous ion activity (7.55 ± 0.10 mg/mL) for FRAP, in the benzene extract of the leaf for FRAP (7.55 ± 0.10 mg/mL), and in the methanol extract of the bark for TAC (2.97 ± 0.01 mg/mL). Tatsimo et al. [73] determined the antioxidant activity of methanol, hexane, ethanol, and butanol leaf extracts of *G. senegalensis* and three bioactive compounds were determined using the FRAP and DPPH free radical–scavenging assays. The results of the radical–scavenging activity using DPPH showed compound **111** with an EC_50_ value of 14.25 ± 1.15 µg/mL while compound **114** had an EC_50_ value of 7.58 ± 0.43 µg/mL and compound **115** with an EC_50_ value of 7.99 ± 0.11 µg/mL. The butanol extracts were the most active among the extracts with an EC_50_ of 26.44 ± 0.56 µg/mL. The ethyl acetate, methanol, and hexane extracts had EC_50_ values of 37.03 ± 0.87 µg/mL, 64.76 ± 0.74 µg/mL, and 78.11 ± 1.19 µg/mL, respectively. The ethyl acetate and butanol extracts from *G. senegalensis* had high reductive abilities compared with the methanol extracts. Compound **111** had the lowest ferric iron-reducing power whereas compounds **114** and **115** had the highest ferric iron-reducing activity at the concentrations tested. Compound **116** did not show any antioxidant activity. Other compounds, such as compounds **109**, **110**, **113**, **117**, and **118** were obtained in small quantities and consequently were not tested for antioxidant activities.

#### 3.6.6. Antidiabetic Activity

Mann et al. [88] investigated the antidiabetic activity of the crude extract and partitioned fractions of *M. senegalensis*. The roots and methanol extracts of *M. senegalensis* stem bark were reported to have a 29.75% reduction in fasting glucose levels compared to a 27.03% reduction when glibenclimide was used after 2 weeks. The herbal preparation ADD-199 (*Maytenus senegalensis*, *Annona senegalensis*, *Kigelia africana*, and *Lannea welwitschii*) was tested for its antidiabetic and antioxidant properties in STZ-induced diabetic C3H mice. The results were compared with those of two allopathic hypoglycemic medications, metformin and glibenclimide [89]. According to the findings, the plasma insulin levels of untreated diabetic mice were at trace levels, while those of normal controls at termination were approximately 76 µmol/L. Metformin did not affect insulin levels, whereas glibenclamide and ADD-199 raised them in diabetic mice by up to 70%, compared to untreated non-diabetic animals. In less than two weeks, 100 mg/kg ADD-199 raised the basal plasma glucose levels of diabetic controls from 18.8 mM to 14.0 mM, whereas metformin and glibenclamide took four and six weeks, respectively. The ADD-199 hypoglycemic effect appeared to be linked to alkaloids and phytochemicals in the extracts. The observed rise in plasma lipids was reversed by treatment with ADD-199 or the hypoglycemic drugs, however, hepatic glycogen, triacylglycerol, and cholesterol levels rose instead. Additionally, the treatment reduced hepatic-lipid peroxidation and enhanced glucose uptake by isolated diaphragms. The antihyperglycemic and antioxidant effects of ADD-199 at a dose of 100 mg/kg/day are like those of metformin (50 mg/kg) and glibenclamide (0.25 mg/kg), the highest daily therapeutic doses.

#### 3.6.7. Antiviral Activity

Hussein et al. [65] screened *M. senegalensis* methanol and aqueous stem bark extracts for their antiviral activity using HIV-induced cytopathic effects (CPE) on MT-4 cells and HIV-1 protease activity. The stem bark had considerable inhibitory effects against HIV-1 protease activity with an IC_50_ value of 125 mg/mL. The water and methanol extracts had moderate efficacy against HIV-1 protease with 56.8% and 48% inhibition and IC_50_ values of 88 µg/mL and 105 µg/mL, respectively. The water-soluble fractions had higher inhibitory activity (42.5 ± 3.4%–68.9 ± 4.5%) than the ethyl acetate soluble fraction (25.3 ± 3.7%). Several compounds isolated compounds from the stem bark of *M. senegalensis* including ((−)-4′-methylepigallocatechin (**1**), (−)-epicatechin (4β→8) (−)-4′-methylepigallocatechin (**6**), and phloroglucinol-1-O-β-D-glucopyranoside (**8**), displayed interesting anti-HIV protease activity with inhibitory activity ranging from 68.2 to 72.9%. Other isolated compounds, including ((−)-4′-methylepigallocatechin (**2**), (−)- epigallocatechin (**3**), epicatechin (4β→8) epigallocatechin (**5**), (−)-epicatechin (4β→8) (−)-4′-methylepigallocatechin (**6**), (−)-epicatechin (4β→8) (−)-epicatechin (procyanidin B-2) (**14**), (−)-4′-methylepigallocatechin 5-O-β-D-glucopyranoside (**2**), (.)-4′-methylgallocatechin 3′-O-β-D-glucopyranoside (**4**), and phloroglucinol-1-O-β-D-glucopyranoside (**8**) had anti-HIV-1 protease activity at 100 µM.

#### 3.6.8. Anti-Sickling Activity

Sall et al. [90] determined the antisickling potential of *M. senegalensis* methanolic and ethyl acetate extracts on SS sickle types. The methanol extracts had a good antisickling effect with a maximum antisickling reverse of 72% for *M. senegalensis* at 10 mg/mL in 120 min of incubation while the ethyl acetate extract at the same conditions showed a sickling reverse of 62%.

#### 3.6.9. Toxicology Studies on *M. senegalensis* and Isolated Compounds

Nyarko et al. [91] determined the sub-chronic toxicity of the aqueous antidiabetic herbal extract ADD-199, prepared from *Maytenus senegalensis*, *Annona senegalensis*, *Kigelia africana*, and *Lannea welwitschia* combination in male Wistar albino rats. ADD-199 herbal extract was administered at a daily dose of 100 mg/kg or 500 mg/kg body weight for 30 days. ADD-199 did not affect plasma levels of albumin or creatinine kinase (CK), alkaline phosphatase (ALP), alanine aminotransferase (ALT), or aspartate aminotransferase (AST). Additionally, it did not affect urea and plasma creatinine levels. Furthermore, packed cell volume and blood hemoglobin, red blood cells, reticulocytes, platelets, lymphocytes, and granulocyte levels were unaffected by ADD-199. However, at day 15, it resulted in notable dose-dependent decreases in white blood cell counts, with varied levels of recovery by day 30. After week three, it also slowed the rate at which body weight increased. However, organ weights at termination showed no alterations. Pentobarbital-induced sleep durations, zoxazolamine-induced paralysis, and specific cytochrome P450 isozyme activities in rats were not substantially impacted by ADD-199. Malebo et al. [32] investigated the acute toxicity effects of crude *M. senegalensis* root bark ethanolic extract in mice. At all the tested dosages of 200 mg/kg, 300 mg/kg, 400 mg/kg, 800 mg/kg, and 1600 mg/kg body weight, no mouse died at 24 hrs and 48 hrs post extract administration, and no toxic signs were seen in the mice at all the doses tested. The therapeutic index was estimated to be higher than 113.5, indicating that *M. senegalensis* ethanol extract is not toxic. The ethanol extract of *M. senegalensis* was shown to be non-toxic when administered orally at the tested dosages, and the lethal dose was higher than 1600 mg/kg body weight since there was no mortality recorded. Murjanatu et al. [79] investigated the acute toxicity effects of the leaf methanolic extract of *M. senegalensis* via the intraperitoneal route using mice and rats. The methanolic leaf extract had a median lethal dose (LD_50_) of 1264.91 mg/kg in both rats and mice. The LD_50_ indicated the leaf extract to be slightly toxic to the experimental models. Umar et al. [28] evaluated the safety profile of *M. senegalensis* methanol root extract on mice. The acute toxicity study (10 mg/kg, 100 mg/kg, 1000 mg/kg bw of the extract) and the sub-chronic toxicity study (1600 mg/kg, 2900 mg/kg, and 5000 mg/kg bw of the extract) did not result in any mortality among the experimental animals. Therefore, the LD_50_ was >5000 mg/kg bw for the acute toxicity. All the extract doses examined in the sub-chronic toxicity research substantially increased (*p* < 0.05) the serum concentrations of creatinine, urea, and alkaline phosphatase (ALP), in comparison to the control group. However, the extracts did not significantly reduce (*p* > 0.05) the concentration of sodium potassium, chloride, alanine transaminases, and aspartate transaminases in the serum of the tested animals as compared to the reference standard used in the study. The white blood cell (WBC) count was considerably (*p* < 0.05) higher in the rats given 400 and 800 mg/kg bw methanol root extracts when compared to the control group. The platelet count was considerably (*p* < 0.05) greater in rats given 800 mg/kg bw. There was negligible difference in packed cell volume, red blood cell, and hemoglobin in the groups treated with 200 mg/kg, 400 mg/kg, and 800 mg/kg bw of *M. senegalensis* root extract compared to the control group. Jigam et al. [23] tested the sub-chronic toxicity of purified fraction of *M. senegalensis* leaf extracts. Sub-chronic administration of the purified fraction of *M. senegalensis* leaf extracts significantly (*p* < 0.05) increased the concentrations of aspartate transaminase and alanine transaminase, and proteins. However, the fraction had negligible effect (*p* < 0.05) on sodium, potassium, chloride, alkaline phosphatase, triglyceride, and glucose concentrations. Sanda et al. [63] determined the acute toxicity study of *M. senegalensis* ethanol leaf extract using mice. There was no animal mortality at the highest dose administered. Moreover, other toxicity signs such as depression and anorexia were observed. Da Silva et al. [13] used adult male CD-6 mice to assess the acute toxicity of *M. senegalensis* leaf and stem extracts. At the same tested dosage of 1200 mg/kg of body weight, *M. senegalensis* stem extract was toxic while the leaf extract showed some toxicity. The acute and subacute toxicity of the polar fraction and the crude methanol extract of the root bark of *M. senegalensis* were examined [24]. The rats treated with the crude methanol extract and polar fraction up to 5000 mg/kg did not exhibit any signs of mortality or health decline, indicating that the LD_50_ in rats is higher than 5000 mg/kg. Neither the 24-h extract administration period nor the two-week observation period resulted in any of the rats losing more than 10% of their body weight. However, within hours after extract administration, rats treated with 2900 mg/kg and 500 mg/kg crude methanol extract showed signs of restlessness, hyperactivity, and deep breathing, although no mortality was observed. These symptoms also persisted longer in mice treated with 1600 mg/kg, 2900 mg/kg, and 5000 mg/kg of the polar fraction than in rats treated with crude methanol extract. Nevertheless, rats administered both the crude methanol extract and the polar fraction at dosages of ≤1000 mg/kg showed no effects. Both the crude methanol extract and the polar fraction were found to have a maximum tolerable dose of 1000 mg/kg. When male rats were given crude methanol extract at 800 mg/kg and the polar fraction at 200 and 300 mg/kg for 21 days, their serum levels of aspartate transaminase, alanine transaminase, alkaline phosphatase, and total protein concentration increased in a dose-dependent manner in comparison to the control rats. The serum of animals treated with 800 mg/kg of crude methanol extract and 200 mg/kg and 300 mg/kg of the polar fraction showed a significant increase (*p* < 0.01) increase in sodium and potassium and a decrease in urea levels. The alterations in biochemical markers were reversed 2 weeks after discontinuation of extract intake. The results strongly indicated that various serum indicators of hepato-renal integrity in male rats were temporarily altered by subacute administration of the crude methanol extract and polar fraction. Verschaeve and Van Staden [92] investigated in vitro genotoxic and antigenotoxic activity of *M. senegalensis* methanol and dichloromethane root-bark extracts using the bacterial Ames, Umu-C, and VITOTOX^®^ tests, and with the cytochalasin B micronucleus test and alkaline comet assay in human white blood cells. Three *Salmonella typhimurium* strains, TA 98 (which identifies frame-shift mutations), TA 100 (which identifies base-pair substitutions), and TA 102 (which detects oxidative and DNA cross-linking damage) were used without metabolic activation to determine genotoxicity. The addition of the S9 microsomal fraction mimics the metabolism of test chemicals in mammals. The Ames test, with and without S9 in strains TA 98 and TA 100, VITOTOX^®^ test, with and without S9, Umu-C test, with and without S9, micronucleus test in human white blood cells, without S9, and alkaline comet assay in human white blood cells, without S9, were all used. The DCM extracts of *G. senegalensis* were found to be mutagenic in the Umu-C test in the absence of S9, which was in contradiction with the results obtained for the Ames and VITOTOX^®^ tests. Furthermore, in the micronucleus test, DCM extracts of *G. senegalensis*-induced micronuclei were found in human white blood cells. Because DCM extracts had no effect in other tests without a spindle apparatus, the activity was attributed to possible interaction with the apparatus.

#### 3.6.10. Clinical Trials

The first clinical trial assessing the safety and tolerability of *M. senegalensis* ethanol root-bark extracts was carried out in Tanzania by Kassimu et al. [33] on healthy adult male volunteers. The extracts were administered at increasing doses (400 mg, 600 mg, and 800 mg every 8 h for four days). There were no fatalities, severe adverse events, or adverse events deemed clinically significant that led to the discontinuation of the tested product or the termination of the trial. In addition, no clinical adverse events, either solicited or unsolicited, were recorded within the first 28 days after the tested medicine was administered. All the adverse events that were reported were laboratory-related and were assigned a severity rating of either 1 or 2. Upon clinical analysis of vital signs, 12-lead electrocardiogram data, and physical examination, no safety warning signs were found. The study findings suggested the potential immunomodulatory effects of the products tested. Kassimu et al. [34] recently conducted clinical trials in Tanzania, to assess the safety and tolerability of the anti-malarial herbal remedy of *M. senegalensis* in healthy adult Tanzanian volunteers aged from 18 to 45 years. The trial aimed to specifically evaluate the electrocardiographic effects of *M. senegalensis* in healthy human adult volunteers. Results from a group of 12 healthy people show that different dosages of *M. senegalensis* extract did not cause a discernible extension of the QT interval, which represents electrical depolarization (contraction) and repolarization (recovery) of the ventricles. These findings confirm the overall safety profile of *M. senegalensis* by highlighting its advantageous cardiac profile. There were no adverse QTcF (QT corrected for heart rate by Fridericia’s cube root formula) occurrences with any dosage tested. On day 28, the only participant who received the maximum dosage of *M. senegalensis* (800 mg) changed somewhat from baseline. For each of the four study dose groups, the volunteers’ maximum QTcF and maximum △QTcF were consistent. The electrocardiographic parameters of healthy volunteers were unaffected by a four-day course of 800 mg of *M. senegalensis* every eight hours. The results of the study validated both the traditional use and the modern therapeutic potential of *M. senegalensis*. They also laid the basis for future studies that examine the safety profile of *M. senegalensis* in various demographic groupings, involving bigger and more varied populations considering the extensive usage of *M. senegalensis* in traditional medicine and its potential as a therapeutic agent.

**Table 3:** Pharmacological activities of secondary metabolites isolated from *M. senegalensis*.

Compounds	Pharmacological Activities	Bioassay Method	Results	References
Maytenoic acid (**9**)	Antibacterial Anti-inflammatory	Serial dilution In vivo study on mice	MIC = 195 µg/mL against *S. aureus*. Antiphlogistic effects effect (ID_50_ = 0.11 µmol/cm^2^) and oedema inhibition	[20,21]
Pristimerin (**24**)	Antiproliferative Antiplasmodial	Cytotoxicity (human peripheral blood lymphocyte) In vitro test against *P. falciparum* (Dd2)	IC_50_ of 6.8 ± 0.8 μg/mL. They had an IC_50_ of 0.5 μg/mL.	[65]
Maysedilactone (**10**)	Cytotoxicity	MTT (Mouse lymphoma cell line)	<10% inhibition at 10 µg/mL	[16]
9,10-Dihydroxy-4,7-megastigmadien-3-one (**11**)	Cytotoxicity	MTT (Mouse lymphoma cell line)	<20% inhibition at 10 µg/mL	[16]
(−) Epicathechin (**12**)	Cytotoxicity	MTT (Mouse lymphoma cell line)	<20% inhibition at 10 µg/mL	[16]
(+) Gallocathechin (**13**)	Cytotoxicity	MTT (Mouse lymphoma cell line)	30% inhibition at 10 µg/mL	[16]
(−) Epigallocathechin (**3**)	Cytotoxicity Antiviral	MTT (Mouse lymphoma cell line). HIV-1 PR assay	100% inhibition at 10 µg/mL. 43.6% ± 4.5 inhibition of Anti-HIV-1-protease.	[16,65]
Procyanidin B-2 (**14**)	Cytotoxicity Antiviral	MTT (Mouse lymphoma cell line). HIV-1 PR assay	No inhibition of the lymphoma cells at the concentration tested. Had anti-HIV-1-protease activity of 59% ± 2.3	[16,65]
2,3-Dihydrokaempferol 3-O-β-D-glucopyranoside (**15**)	Cytotoxicity	MTT (Mouse lymphoma cell line)	No inhibition at the conc tested	[16]
Quercetin 3-O-β-D-glucopyranoside (**16**)	Cytotoxicity	MTT (Mouse lymphoma cell line)	<30% inhibition at 10 µg/mL	[16]
Kaempferol 3-O-β-D-xylopyranoside (**17**)	Cytotoxicity	MTT (Mouse lymphoma cell line)	<30% inhibition at 10 µg/mL	[16]
Quercetin 3-O-β-D-xylopyranoside (**18**)	Cytotoxicity	MTT (Mouse lymphoma cell line)	<40% inhibition at 10 µg/mL	[16]
3,5-Dimethylgallate (**19**)	Cytotoxicity Antimicrobial	MTT Serial dilution	<10% inhibition of Mouse lymphoma at 10 µg/mL. Had cell viability of >70% against MKN45 cells at 50 µM. No bacterial growth inhibition at 50 µM.	[16,72]
Lupenone (**20**)	Anti-inflammatory	In vivo study on mice	Decreased oedema at 0.1 µmol/cm^2^ by 26% and 65% at 1 µmol/cm^2^ dose	[21]
β-Amyrin (**21**)	Anti-inflammatory	In vivo study on mice	Reduced oedema by 19% to 62% at 26% and 65% at the doses tested.	[21]
(−)-4′-Methylepigallocatechin (**1**)	Antiviral	HIV-1 PR assay	Had anti-HIV-1-protease activity with 34.6% ± 0.8 inhibition	[65]
(−)-4″-Methylepigallocatechin 5-*O*-β-glucopyranoside (**2**)	Antiviral	HIV-1 PR assay	Had good anti-HIV-1-protease with 72.9% ± 4.5 inhibition	[65]
(+)-4″-Methylgallocatechin 3″-*O*-β-glucopyranoside (**4**)	Antiviral	HIV-1 PR assay	Exhibited 68.2% ± 5.3 inhibitory activity against HIV-1-protease	[65]
Epicatechin (4β→8) epigallocatechin (**5**)	Antiviral	HIV-1 PR assay	Had only 33.3% ± 5.9 inhibition	[65]
(−)-Epicatechin (4β→4) (−)-4 0-methylepigallocatechin (**6**)	Antiviral	HIV-1 PR assay	Demonstrated anti-HIV-protease inhibition of 30.5% ± 13.5.	[65]
Phloroglucinol 1-*O*-*β*-D-glucopyranoside (**8**)	Antiviral	HIV-1 PR assay	Had interesting anti-HIV-protease activity of 68.2% ± 4.2	[65]
(2S)-1-O-(4′Z,7′Z,10′Z-Octadecatrienoyl) glycerol (**94**)	Antimicrobial Antiproliferative	Serial dilution MTT	No bacterial growth inhibition at 50 µM. Decreased cell viability to 67% against MKN45 cells	[72]
(2R)-Methyl [(6′-O-galloyl)-β-D-glucopyranosyloxy] phenylacetate (**95**)	Antimicrobial Antiproliferative	Serial dilution MTT	No bacterial growth inhibition at 50 µM. Had cell viability of >70% against MKN45 cells.	[72]
(S)-6′-O-Galloylsambunigrin (**96**)	Antimicrobial Antiproliferative	Serial dilution MTT	No bacterial growth inhibition at 50 µM. Had cell viability of >70% against MKN45 cells.	[72]
(R)-6′-O-Galloylprunasin (**97**)	Antimicrobial Antiproliferative	Serial dilution MTT	No bacterial growth inhibition at 50 µM. Had cell viability of >70% against MKN45 cells	[72]
1-O-β-D-(6′-O-Galloyl)-glucopyranosyl-3-methoxy-5-hydroxybenzene (**98**)	Antimicrobial Antiproliferative	Serial dilution MTT	No bacterial growth inhibition at 50 µM. Cell viability of >70% against MKN45 cells.	[72]
Quercetin (**99**)	Antimicrobial Antiproliferative	Serial dilution MTT	No bacterial growth inhibition at 50 µM. Had relatively weak to moderate decrease in viability against DLD1, MCF7 and MKN45 cancer cell lines.	[72]
Kaempferol 3-O-α-L-arabinofuranoside (**100**)	Antimicrobial Antiproliferative	Serial dilution MTT	No bacterial growth inhibition at 50 µM. Had decrease of cell viability to 68% against MKN45 cells	[72]
Quercetin 3-O-α-L-arabinofuranoside (**101**)	Antimicrobial Antiproliferative	Serial dilution MTT	No bacterial growth inhibition at 50 µM. Relatively weak to moderate decrease in cell viability against DLD1, MCF7, and MKN45 cancer cell lines.	[72]
Kaempferol 3-O-α-L-rhamnopyranoside (**102**)	Antimicrobial Antiproliferative	Serial dilution MTT	No bacterial growth inhibition at 50 µM. Had 68.4% cell viability against MCF-7 cells at 50 µΜ.	[72]
Quercetin 7-O-α-L-rhamnopyranoside (**103**)	Antimicrobial Antiproliferative	Serial dilution MTT	No bacterial growth inhibition at 50 µM. Had cell viability of >70% against MKN45 cells	[72]
Quercetin 3-O-β-D-xylopyranoside (**104**)	Antimicrobial Antiproliferative	Serial dilution MTT	No bacterial growth inhibition at 50 µM. Cell viability of >70% against MKN45 cells	[72]
Quercetin-3-O-(6″-galloyl)-β-D-glucopyranoside (**105**)	Antimicrobial Antiproliferative	Serial dilution MTT	No bacterial growth inhibition at 50 µM. Cell viability of >70% against MKN45 cells at 50 µM.	[72]
Epicatechin 3-O-gallate (**106**)	Antimicrobial Antiproliferative	Serial dilution MTT	No bacterial growth inhibition at 50 µM. Had cell viability of >70% against MKN45 cells at 50 µM.	[72]
Epigallocatechin-3-O-gallate (**107**)	Antimicrobial Antiproliferative	Serial dilution MTT	No bacterial growth inhibition at 50 µM. Cell viability of >70% against MKN45 cells at 50 µM.	[72]
1:1 isomeric mixture of hesperetin 3′-O-β-D-Glucopyranoside (**108**)	Antimicrobial Antiproliferative	Serial dilution MTT	No bacterial growth inhibition at 50 µM. Decrease of cell viability to 69.6% against DLD1, 65.6% against MCF7, and 55.7% against MKN45 cancer cells.	[72]
β-Sitosterol (**22**)	Antimicrobial Antiproliferative	Serial dilution MTT	No bacterial growth inhibition at 50 µM. Weak to moderate decrease in viability against DLD1, MCF7, and MKN45 cancer cell lines.	[72]
β-Sitosterol glucoside (**109**)	Antimicrobial Antiproliferative	Serial dilution MTT	No bacterial growth inhibition at 50 µM. Decrease of cell viability by 59.0% against MKN45 and >70% cell viability against DLD1 and MCF-7 cells.	[72]
(3R*,5S*,6R*,7E,9ξ)-7-Megastigmene-3,6,9-triol-3-O-β-D-(6′-O-galloyl)glucopyranoside (**110**)	Antimicrobial	Serial dilution	MIC of 64 µg/mL against *P. aeruginosa* and *C. neoformans*.	[73]
1-O-β-D-(6′-O-Galloyl)-glucopyranosyl-3-methoxy-5-hydroxybenzene (**111**)	Antimicrobial	Serial dilution	MIC value of 16 µg/mL against *S. aureus*	[73]
2,6-di-O-Galloyl-β-D-glucose (**112**)	Antimicrobial Antioxidant	Serial dilution DPPH and FRAP	MIC = 32 µg/mL against *P. aeruginosa* and *C. neoformans*. EC_50_ value of 14.25 ± 1.15 µg/mL on DPPH and had the lowest ferric iron reducing power.	[73]
Phaeophytin A (**113**)	Antimicrobial	Serial dilution	MIC value of 16 µg/mL against all tested bacteria and fungi, except against *Shigella flexneri* with MIC value of 32 µg/ml	[73]
Phaeophorbide-a (**114**)	Antimicrobial Antioxidant	Serial dilution DPPH and FRAP	MIC values ranging from 16 µg/mL to 32 µg/mL against all tested bacteria and fungi. Had EC_50_ value of 7.58 ± 0.43 µg/mL on DPPH and high ferric iron reducing activity.	[73]
Chlorine e_6_ trimethyl ester (**115**)	Antimicrobial Antioxidant	Serial dilution DPPH and FRAP	MIC values ranging from 32 µg/mL to 64 µg/mL against all tested bacteria and fungi.Had EC_50_ value of 7.99 ± 0.11 µg/mL and high ferric iron reducing activity.	[73]
(4Z,7Z,10Z)-Octadecatrienoic acid (**116**)	Antimicrobial Antioxidant	Serial dilution DPPH and FRAP	Weak antimicrobial activity against all bacteria and fungi. No antioxidant activity.	[73]
Procyanidin B2 3,3′-di-O-gallate (**118**)	Antimicrobial	Serial dilution	MIC of value of 16 µg/mL against *S. aureus aeruginosa* and *C. neoformans*.	[73]

## 4. Discussions and Future Perspectives

*M. senegalensis*, which is distributed across tropical Africa, from Senegal to Eritrea, southern Africa, and Madagascar, is perhaps one of the most widely used medicinal plants in Africa. Throughout the African continent, the different parts of *M. senegalensis* have been used traditionally to cure a variety of ailments, including cancer, TB, venereal illnesses, inflammation-related disorders, and malaria. Several studies have determined the qualitative and quantitative presence of phytochemicals in *M. senegalensis*, and phytochemical components found include triterpenes, anthraquinones, coumarins, saponins, phenol, alkaloids, flavonoids, tannins, and glycosides. In vitro antiviral, antimicrobial, antiparasitic, antidiabetic, anti-inflammatory, antiproliferative, anti-sickling, and antioxidant activities of *M. senegalensis* on different parts of the plant fractions have been observed and several isolated compounds reported, justifying the traditional use of this plant species as an ethnomedicine. *M. senegalensis* has also been examined for its in vivo sub-chronic toxicity in male Wistar albino rats using ADD-199, an aqueous herbal extract made by combining *Maytenus senegalensis*, *Annona senegalensis*, *Kigelia africana*, and *Lannea welwitschia* and had promising results which further justifies the use of this medicinal plant as a remedy. The acute toxicity effects of crude ethanolic *M. senegalensis* root bark extracts in mice have been widely reported, and it is promising that the crude extracts were not toxic at the doses tested, except in one study where acute toxicity effects of the leaf methanolic extract of *M. senegalensis*, administered via the intraperitoneal route, showed a median lethal dose (LD_50_) of >1000 mg/kg in both rats and mice [79]. The genotoxic and antigenotoxic activities of *M. senegalensis* root bark extracts were also examined in vitro using various assays. The results showed conflicting findings, where the DCM extract was found to be mutagenic in the Umu-C test without S9, but non-mutagenic in the Ames and VITOTOX tests [92]. More studies are required to test the extracts at a wide range of concentrations to establish the lowest concentrations that are non-mutagenic in all the different assays. Further studies to isolate compounds or fractionate the DCM fraction may assist in predicting the biomolecules responsible for the activities from the non-polar fractions. GC-MS or UP-LCMS analysis may assist in predicting the possible biomolecules causing the activities from the non-polar DCM extracts, and more research may be needed to isolate the responsible bioactive compounds or perhaps fractionate the DCM extracts. Of the many biomolecules that were identified from different parts of *M. senegalensis*, only of few compounds were tested for antiplasmodial, anti-inflammatory, and antiproliferative activities, and some in vivo studies were carried out on mice. Isomintlactone (**31**), pristimerin (**24**), and jacareubin (**32**) were the only three compounds that were analyzed for molecular docking. The low yield of isolated compounds makes it challenging to conduct a wide range of pharmacological assays which may explain why fewer compounds have been tested for their pharmacological activities. In addition, the isolation of bioactive compounds using traditional methods like column chromatography is generally very laborious and time-consuming. *M. senegalensis* is one of the very few species which has been investigated for its pharmacological activities from in vitro studies to clinical trials as a potential anti-malarial remedy, which justifies the widespread use of the plant species as an ethnomedicine across the African continent [33,34]. Future human clinical research to investigate the safety and efficacy of *M. senegalensis* should use larger and more diverse populations in other demographic groups and these trials should follow the World Health Organization standards for clinical trials. More in vivo studies are also recommended as premised on the potent antimycobacterial and anti-inflammatory activities observed using *M. senegalensis* crude extracts and fractions.

## 5. Conclusions

This review provides a comprehensive summary of all the studies conducted on phytochemistry, molecular docking, pharmacology, toxicology, ethnopharmacology, botany, and clinical trials on *M. senegalensis*, which is also known as *Gymnosporia senegalensis*. This review reports a total of 119 compounds which were isolated and/or identified from *M. senegalensis* using chromatographic techniques such as GC-MS and HPLC-ESI-MS. The different crude extracts contained phenolic compounds, terpenoids, coumarins, tannins, saponins, alkaloids, anthraquinones, and phlobotanins. Of these isolated compounds, 46 were tested for different pharmacological activities including antiplasmodial, anti-inflammatory, and antiproliferative activities. Isomintlactone (**31**), pristimerin (**24**), and jacareubin (**32**) were further analyzed for their molecular docking and had a strong interaction with prostaglandin E synthase and good binding affinities. The crude extracts and fractions display very promising pharmacological activities, including antiparasitic, antimycobacterial, anti-inflammatory, antiviral, antiproliferative, and antidiabetic, with low toxicity in animal model studies. The safety and tolerability of *M. senegalensis* extract in human trials showed its potential immunomodulatory effects and advantageous cardiac profile. The many pharmacological activities tested, and the human trials results, support the use of this plant species as a traditional medicine.

## Figures and Tables

**Table 1:** Medicinal uses and corresponding parts of *M. senegalensis* in different countries.

Plant Part	Country	Traditional Uses	References
**Bark**	Burkina Faso	Oral diseases, toothache, gingivitis, and sores	[56]
	Ethiopia	Lung cancer, stomach pain.	[3,54]
	Nigeria	Dysentery, ulcers, and wounds	[58]
	Uganda	Syphilis, oral candidiasis	[51]
**Leaves**	Burkina Faso	Malaria, diarrhea, dental pain, headache, oral diseases, toothache, gingivitis, and sores	[56,57]
	Ethiopia	Lung cancer	[3]
	Kenya	Eye infections	[49]
	Nigeria	Dysentery	[58]
	Uganda	Syphilis, oral candidiasis	[51]
	Togo	Diarrhea	[55]
	South Africa	Tuberculosis	[60]
	Zambia	Tuberculosis	[48]
	Zimbabwe	Respiratory ailments including pneumonia and tuberculosis	[47]
		Ocular illnesses, tooth pain, stomatitis, gingivitis, and antibilharzial and anti-ulcerous gastric agents	[44]
**Seeds**	Ethiopia	Epilepsy and headache (In combination with *Ocimum lamiifolium*)	[53]
**Roots**	Senegal	Malaria, fever, diarrhea, and abscess	[40,41,42,43]
	Kenya	Chest pains, rheumatism, snakebites, diarrhea, and fever	[49]
	Botswana	Cough, tuberculosis, and sexually transmitted infections	[45]
	Burkina Faso	Malaria, diarrhea, dental pain, headache, oral diseases, toothache, gingivitis, and sores	[56,57]
	India	Menorrhagia and leucorrhea	[59]
	Nigeria	Stomachache, snakebite, amoebic dysentery, and yellow fever	[58]
	South Africa	Schistosomiasis	[46]
	Uganda	Syphilis, oral candidiasis, and diabetes mellitus	[51,52]
	Zimbabwe	Respiratory ailments including pneumonia and tuberculosis. Schistosomiasis	[46,47]
		Tooth pain, wound skinning, and gonorrhea.	[44]
**Root bark**	Kenya	Malaria	[50]
**Stem bark**	Togo	Diarrhea	[55]
	Sudan	Dysentery, snakebites, and tumors	[10]

## Data Availability

The data presented in this study are available upon request from the corresponding authors.

## Supplementary Materials

The following supporting information can be downloaded at: https://www.mdpi.com/article/10.3390/biom15020197/s1, Structures of the isolated or tentatively identified compounds from *Maytenus senegalensis*/*Gymnosporia senegalensis*.
