# Supplementary material for: A Review of the Phytochemistry, Molecular Docking, Pharmacology, Toxicology, Ethnopharmacology, Botany, and Clinical Studies of Maytenus senegalensis (Lam.) Excell
Source: Biomolecules. 2025 Jan 30;15(2):197. doi: 10.3390/biom15020197 (PMC11853367; doi:10.3390/biom15020197)
Supplement: Supplementary file 1 [file biomolecules-15-00197-s001.zip › biomolecules-3436647-supplementary.pdf]

Structures of the isolated or tentatively identified compounds from *Maytenus senegalensis*/ *Gymnosporia senegalensis*

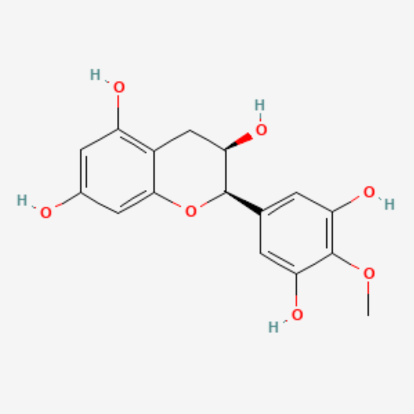

(-)-4′-Methylepigallocatechin **(1)**

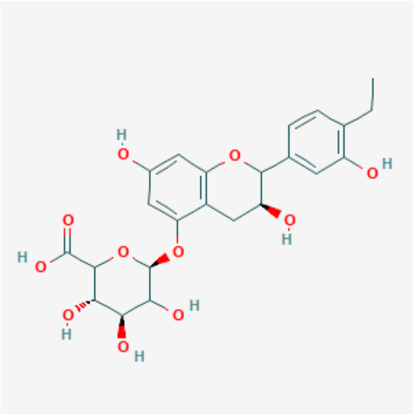

(-)-4″-Methylepigallocatechin 5-*O*-β-glucopyranoside **(2)**

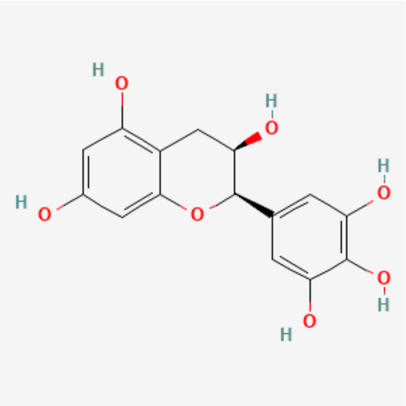

(-)-Epigallocatechin **(3)**

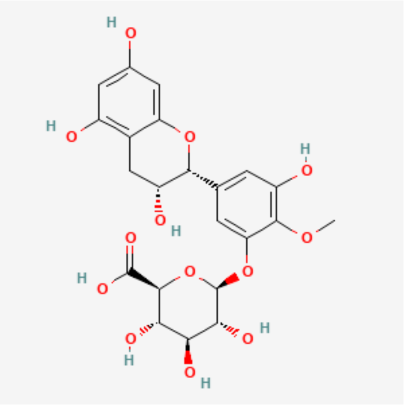

(+)-4″-Methylgallocatechin 3″-*O*-β-glucopyranoside **(4)**

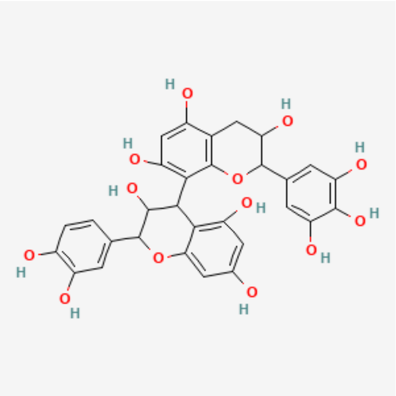

Epicatechin (4β→8) epigallocatechin **(5)**

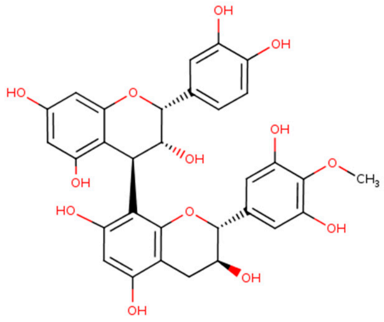

(-)-Epicatechin (4β→4) (-)-4 0-methylepigallocatechin **(6)**

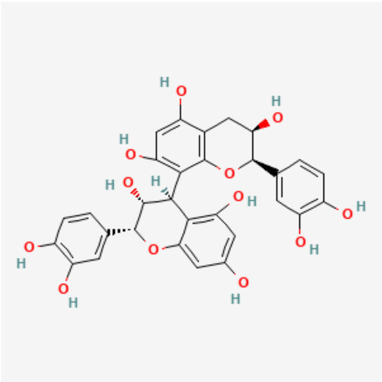

Epicatechin (4β→8) epicatechin **(7)**

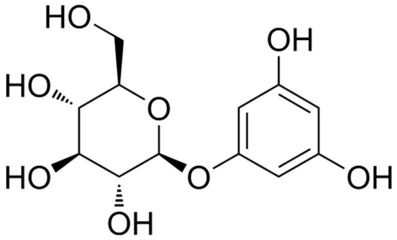

Phloroglucinol 1-*O*-*β*-D-glucopyranoside **(8)**

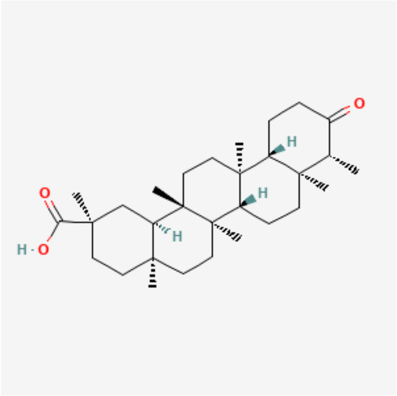

3-Oxo-friedelan-20α-oic acid **(9)**

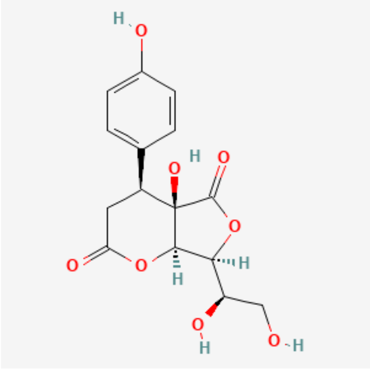

Maysedilactone **(10)**

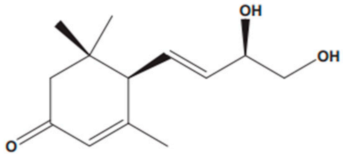

9,10-Dihydroxy-4,7-megastigmadien-3-one **(11)**

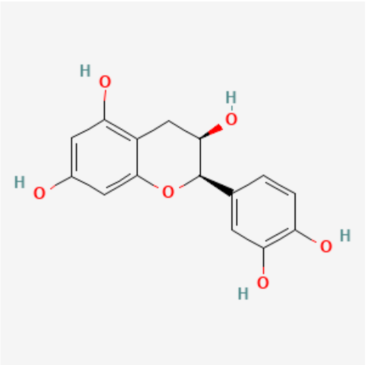

(-) Epicathechin **(12)**

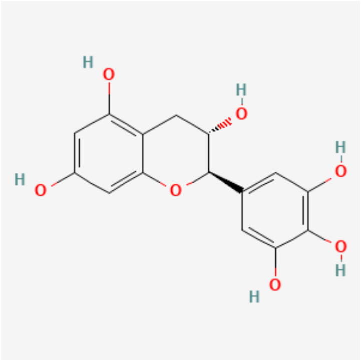

(+) Gallocathechin **(13)**

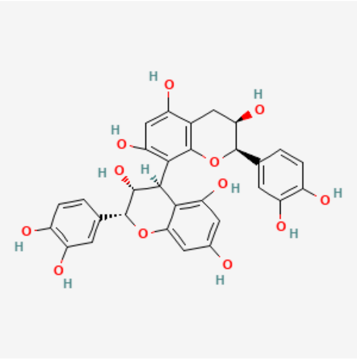

Procyanidin B-2 **(14)**

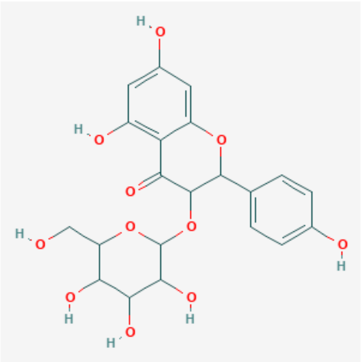

2,3-Dihydrokaempferol 3-O-β-D-glucopyranoside **(15)**

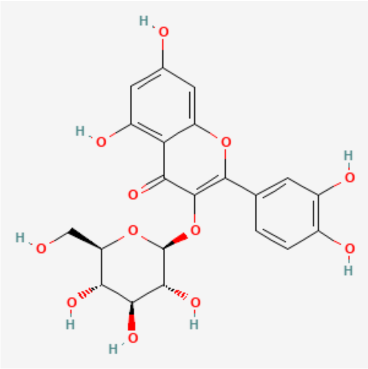

Quercetin 3-O-β-D-glucopyranoside **(16)**

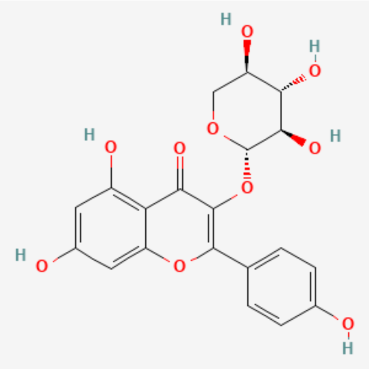

Kaempferol 3-O-β-D-xylopyranoside **(17)**

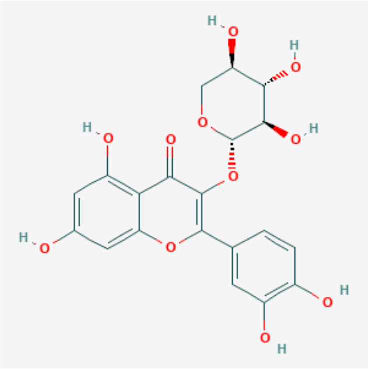

Quercetin 3-O-β-D-xylopyranoside **(18)**

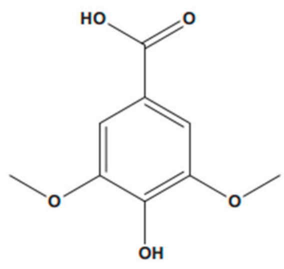

3,5-Dimethylgallate **(19)**

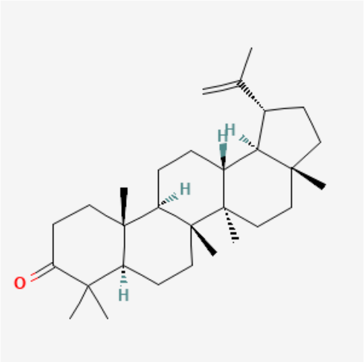

Lupenone **(20)**

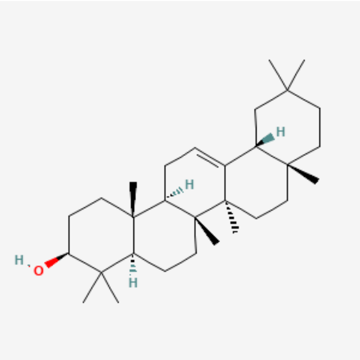

β-Amyrin **(21)**

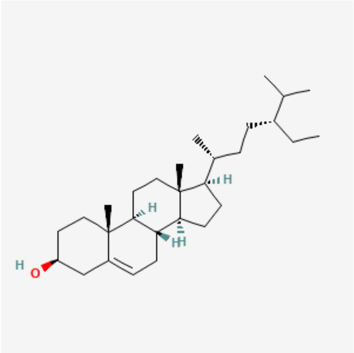

β-Sitosterol **(22)**

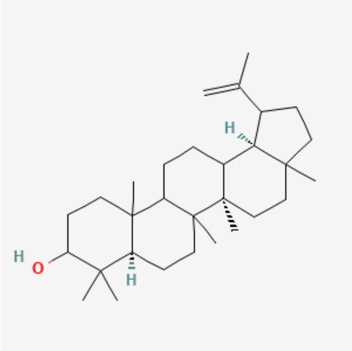

3-Hydroxy-20(29)-lupen-28-ol **(23)**

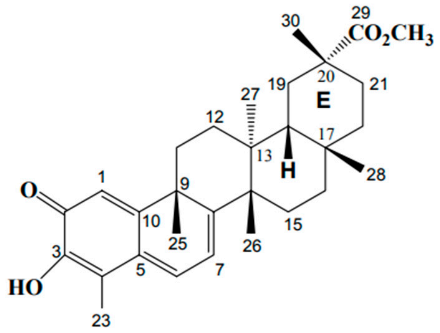

20α)-3-hydroxy-2-oxo-24-nor-friedela-1(10),3,5,7-tetraen-carboxylic acid-(29)-methylester **(24)**

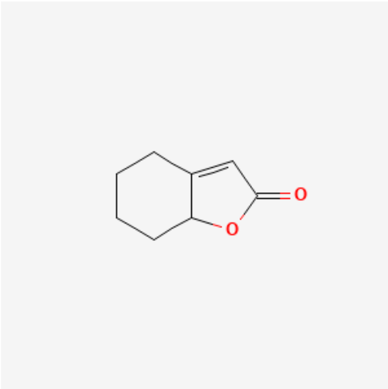

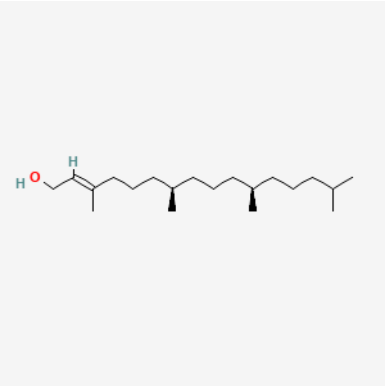

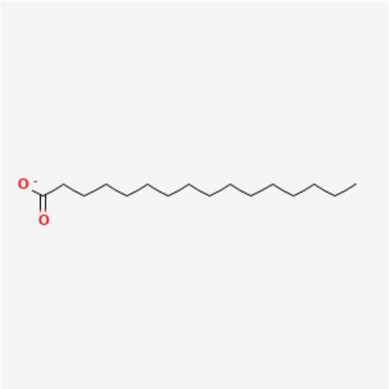

2(4H)-Benzofuranone, 5,6,7,7a-tetrahydro **(25)** Phytol **(26)** *n*-Hexadecanoic acid **(27)**

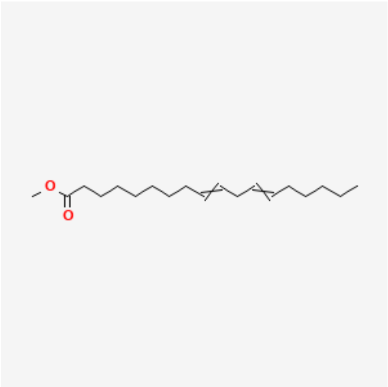

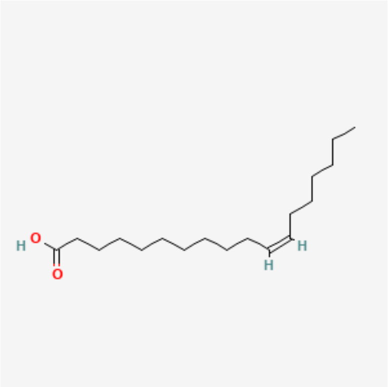

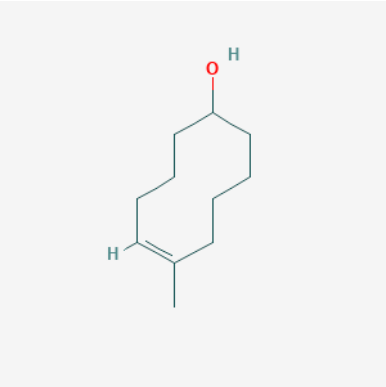

9,12-Octadecadienoic acid, methyl ester **(28)** *cis*-Vaccenic acid **(29)** 6-Methyl-cyclodec-5-enol **(30)**

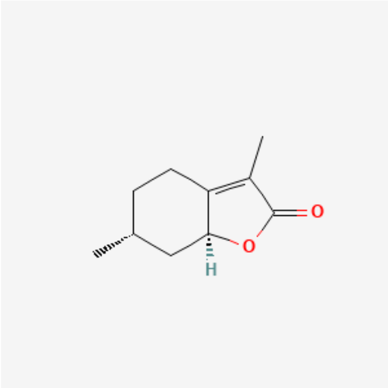

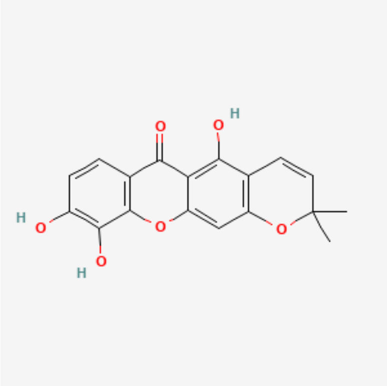

5,6,7,7a-Tetrahydro-2(4H)-benzofuranone **(31)** 2H,6H-pyrano[3,2-b] xanthen-6-one **(32)**

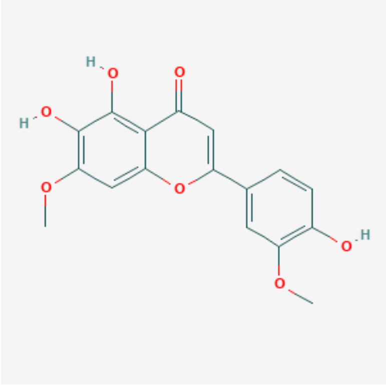

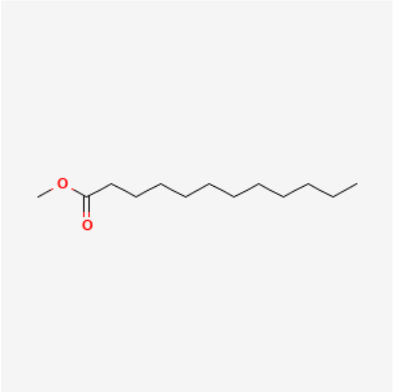

5,7,3′-Trihydroxy-6,4′-dimethoxyisoflavone **(33)** Dodecanoic acid, methyl ester **(34)**

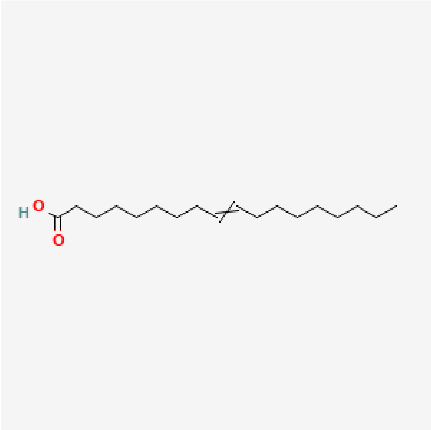

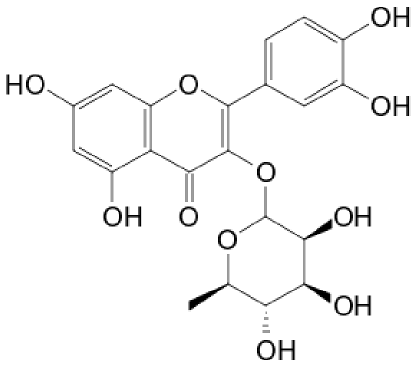

9-Octadecenoic acid **(35)** Quercetin-3-O-dirhamnoside **(36)**

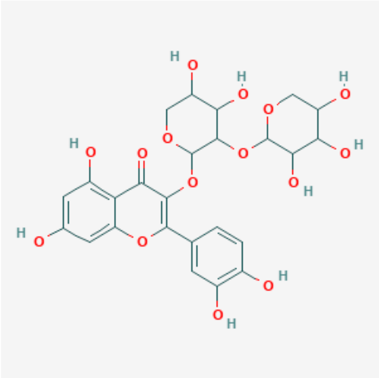

Quercetin-*O*-pentoside **(38)**

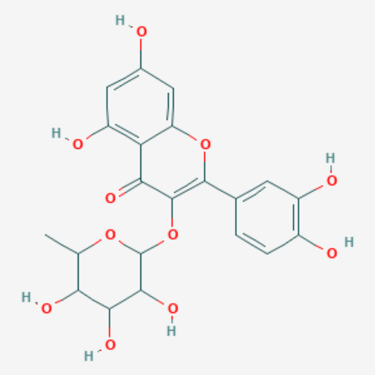

Quercetin-3-*O*-rhamnoside **(40)**

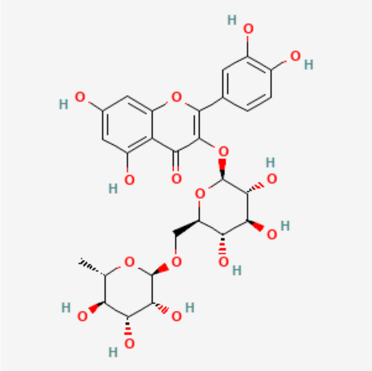

Rutin **(41)**

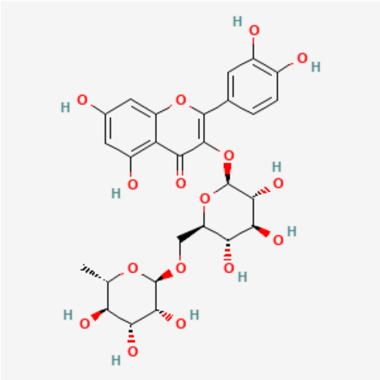

Kaempferol-3-*O*-rutinoside **(43)**

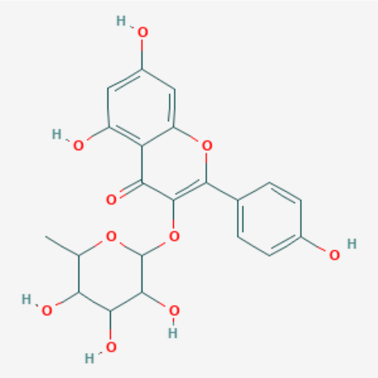

Kaempferol-3-*O*-rhamnoside **(45)**

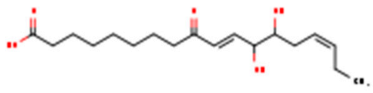

Oxo-dihydroxy-octadecenoic acid **(48)**

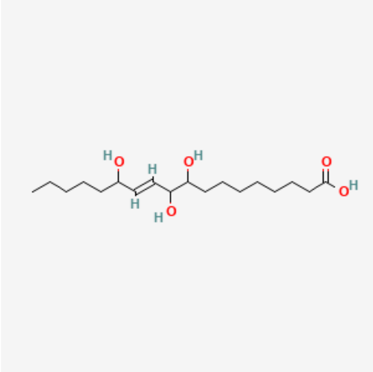

Trihydroxy-octadecenoic acid **(49)**

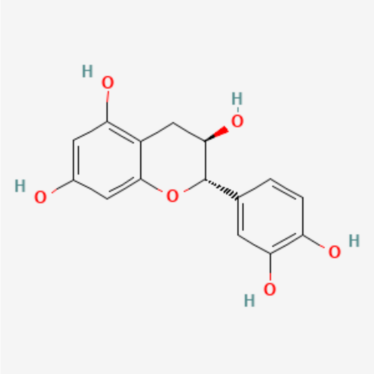

Catechin **(50)**

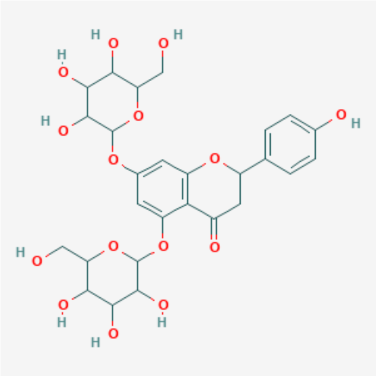

Naringenin-6,8-di-C-hexoside **(51)**

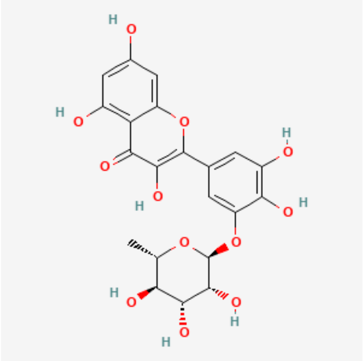

Myricetin-*O*-rhamnoside **(52)**

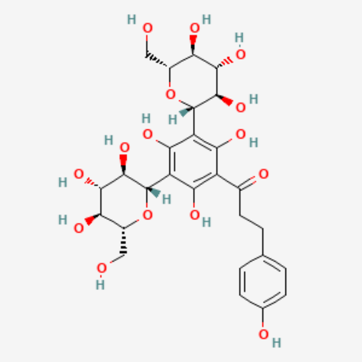

Phloretin-di-Chexoside **(53)**

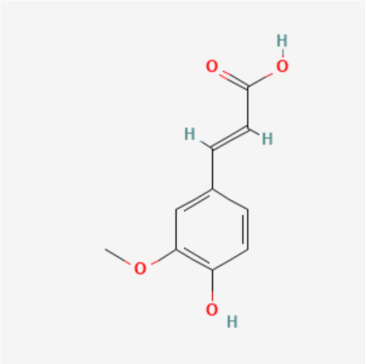

Ferulic acid **(54)**

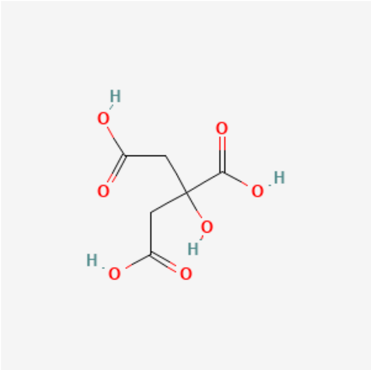

Citric acid **(59)**

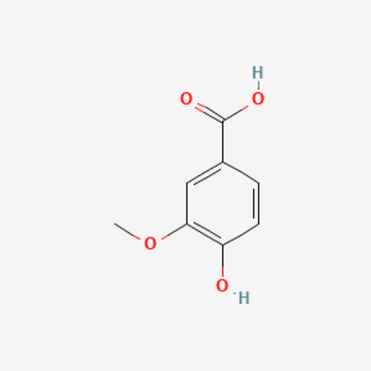

Vanillic acid **(60)**

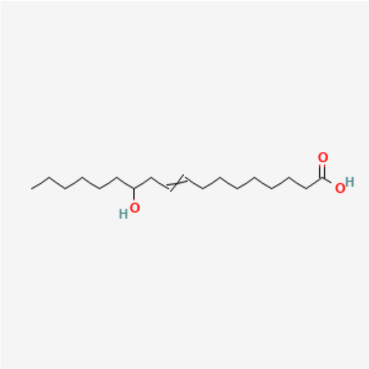

12-Hydroxy-9- octadecenoic acid **(61)**

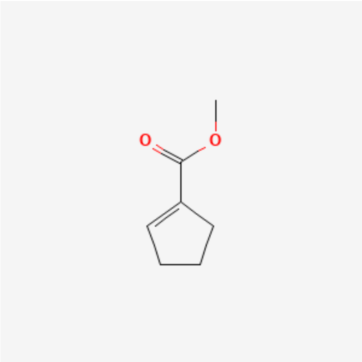

Methyl-1-cyclopentene-1-carbxylate **(62)**

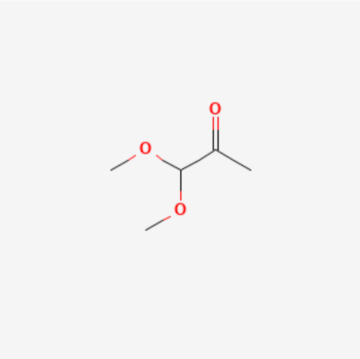

1, 1-Dimethoxyacetone **(63)**

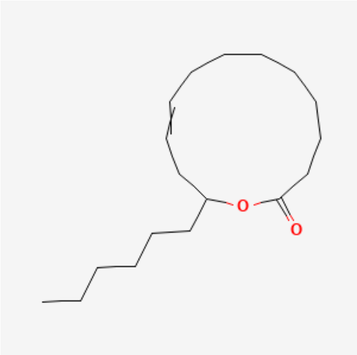

13 hexyloxacyclotridec-10-en-2-one **(63)**

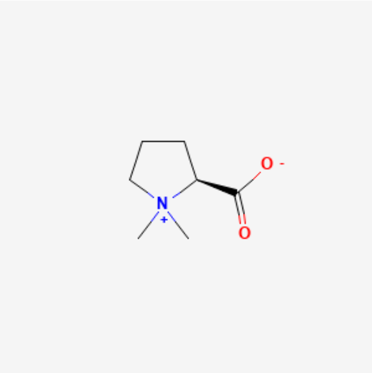

L-Stachydrine **(64)**

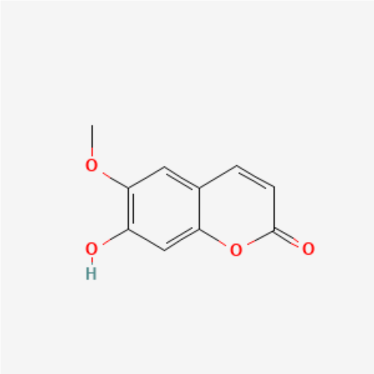

Scopoletin **(65)**

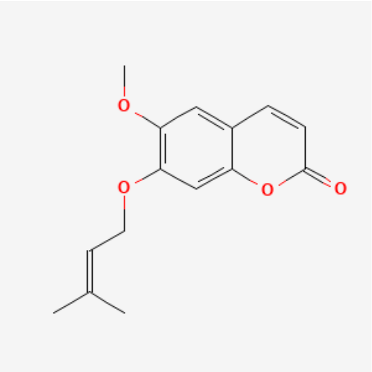

Prenyletin (7-(3′-methyl-2′-butenyloxy)-6-methoxycoumarin) **(66)**

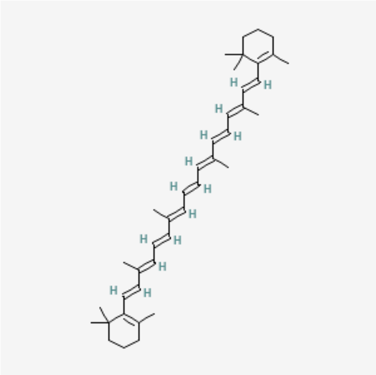

β-Carotene **(67)**

Tetratetracontane **(68)**

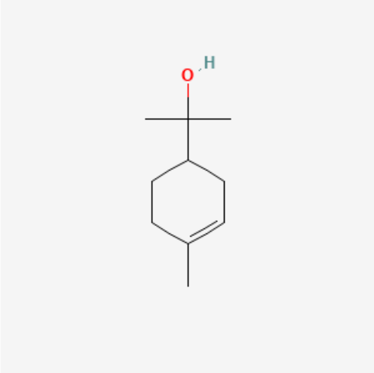

Terpineol **(69)**

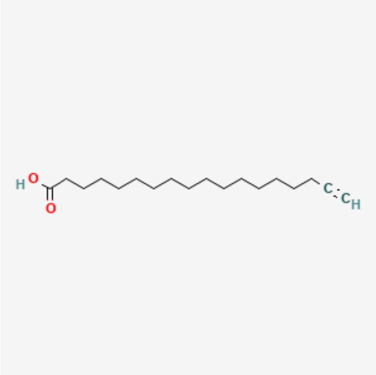

17-Octadecynoic acid **(70)**

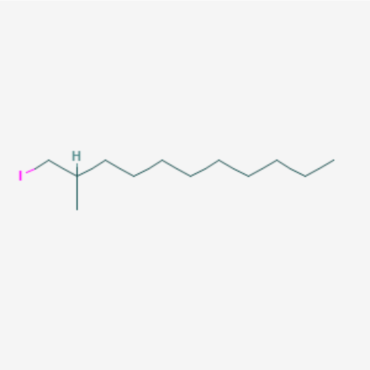

1-Iodo-2-methylundecane **(71)**

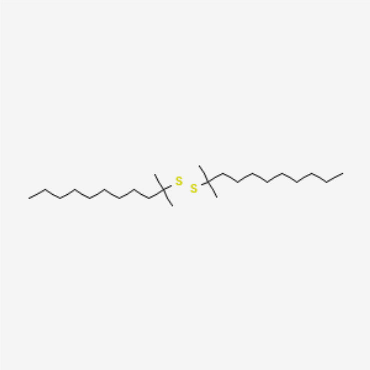

Disulfide, di-tert-dodecyl **(72)**

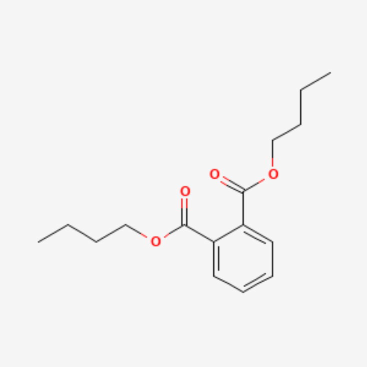

Dibutyl phthalate **(73)**

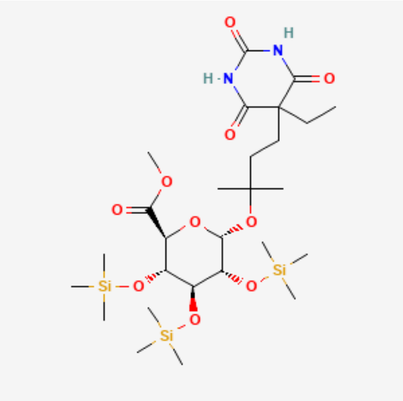

α-D-Glucopyranosiduronic ac-id,3-(5-ethylhexahydro-2,4,6-trioxo-5 pyrimidinyl)-1,1-dimethylpropyl 2,3,4-tris-*O*-(trimethylsilyl)-, methyl ester **(74)**

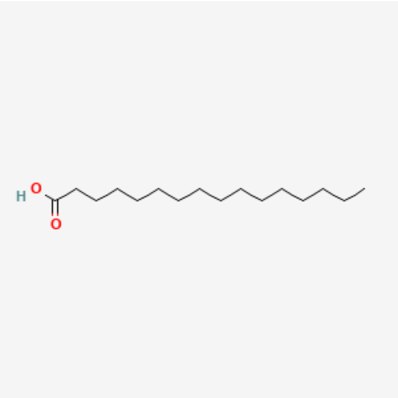

Hexadecaoic acid **(75)**

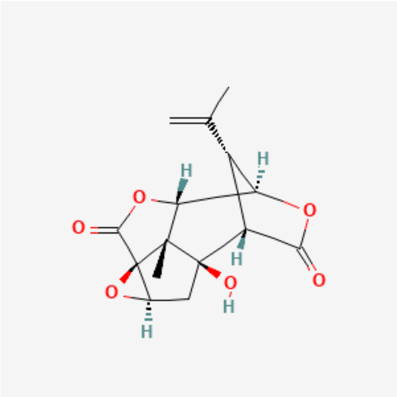

Picrotoxinin **(76)**

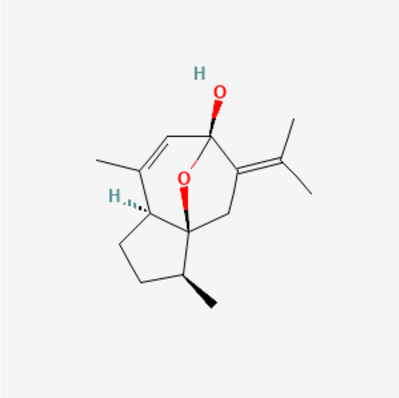

Curcumenol **(77)**

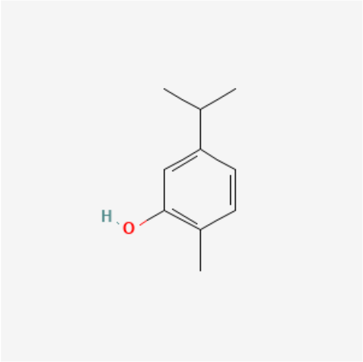

Carvacrol **(78)**

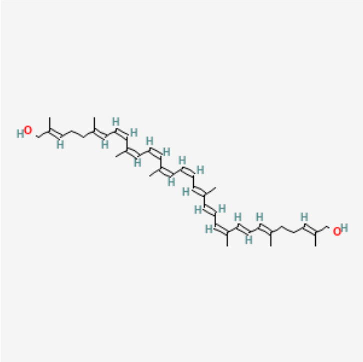

Psi.,.psi.-carotene **(79)**

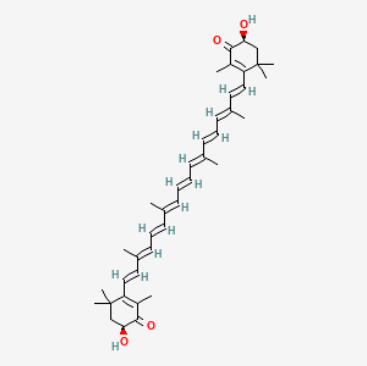

Astaxanthin **(80)**

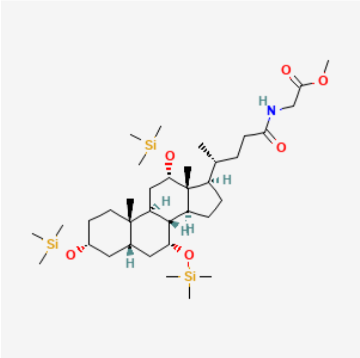

Methyl glycocholate, 3 TMS **(81)**

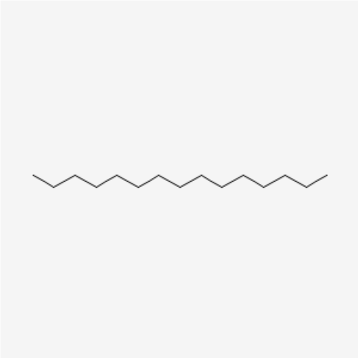

Pentadecane **(82)**

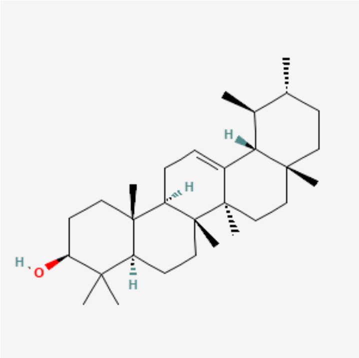

α-Amyrin **(83)**

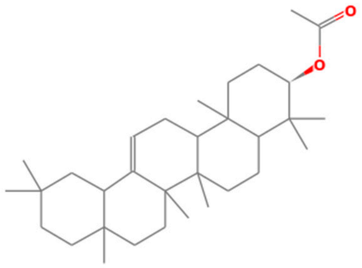

Olean-12-en-3-ol, acetate (3β) **(84)**

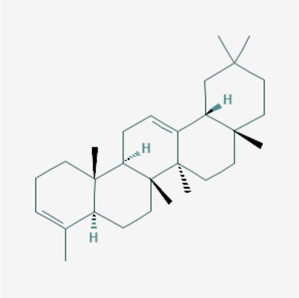

24-Noroleana-3,12-diene **(85)**

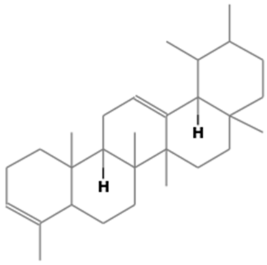

24-Norursa-3, 12-diene **(86)**

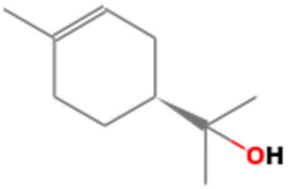

L-α-Terpineol **(87)**

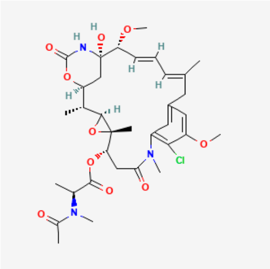

Maytansine **(88)**

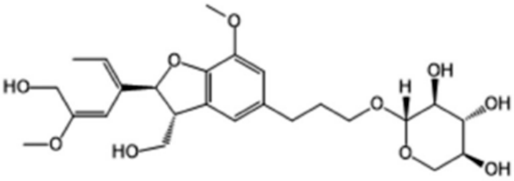

Mayselignoside **(89)**

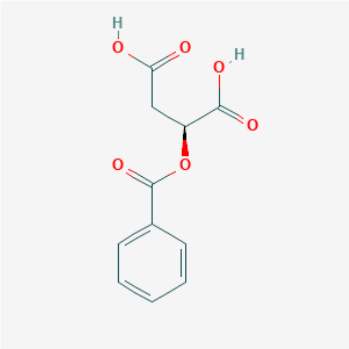

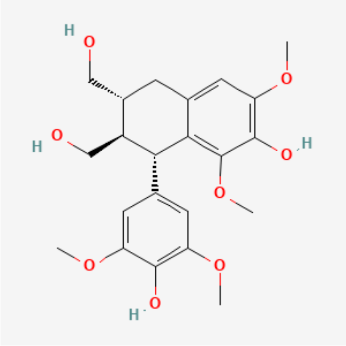

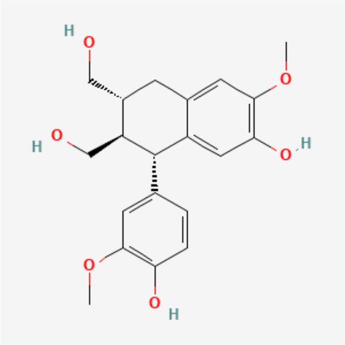

Benzoyl R-(+)-malic acid **(90)** (+)-Lyoniresinol **(91)** (-)-Isolariciresinol **(92)**

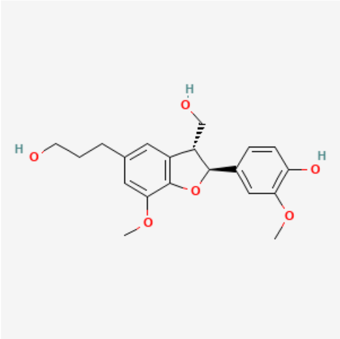

Dihydrodehydrodiconiferyl **(93)**

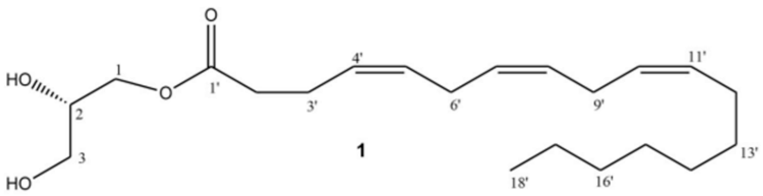

(2S)-1-O-(4′Z,7′Z,10′Z-Octadecatrienoyl) glycerol **(94)**

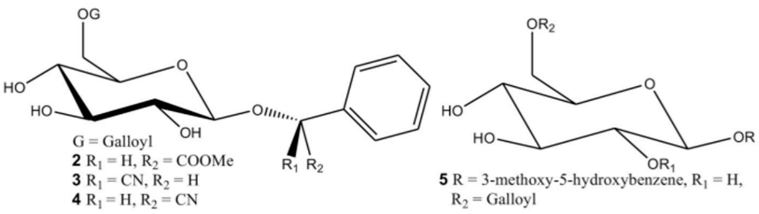

(2R)-methyl [6′-O-Galloyl)-β-D-glucopyranosyloxy] phenylacetate **(95) and** compound **96-98** represented by R_1-R_3**.**

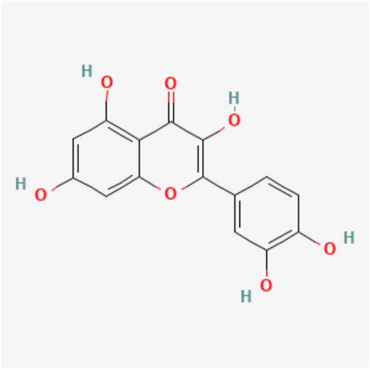

Quercetin **(99)**

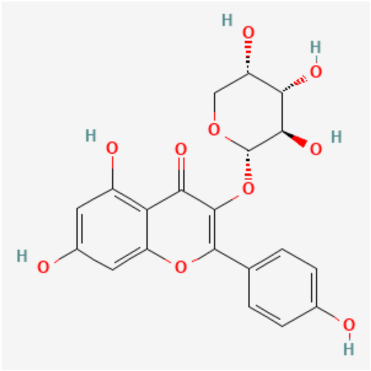

Kaempferol 3-O-α-L-arabinofuranoside **(100)**

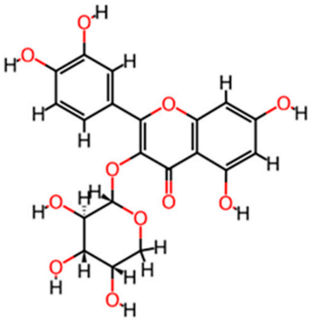

Quercetin 3-O-α-L-arabinofuranoside **(101)**

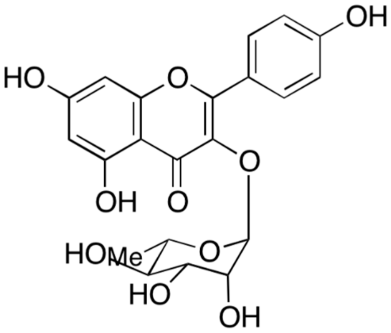

Kaempferol 3-O-α-L-rhamnopyranoside **(102)**

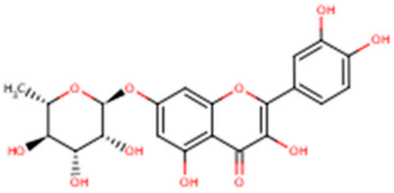

Quercetin 7-O-α-L-rhamnopyranoside **(103)**

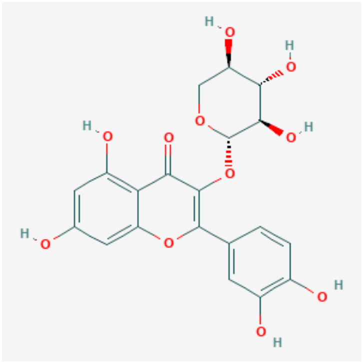

Quercetin 3-O-β-D-xylopyranoside **(104)**

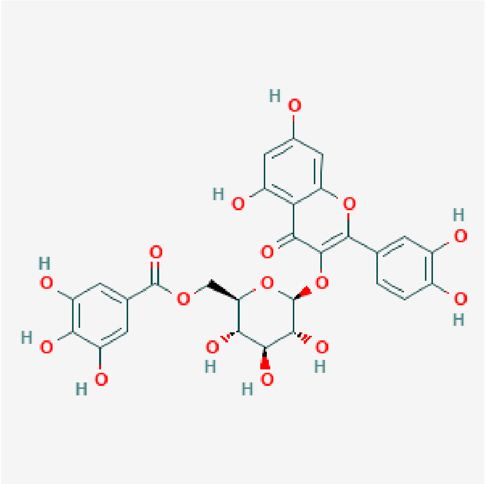

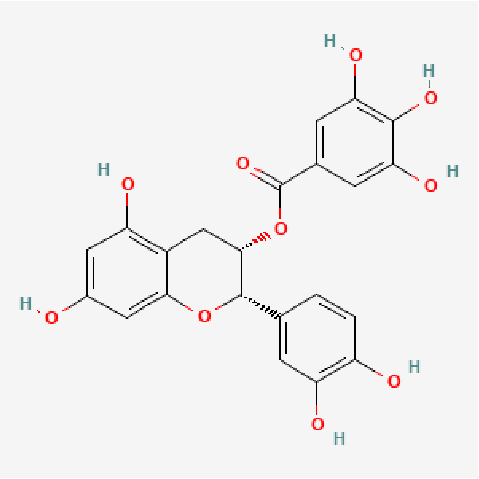

Quercetin-3-O-(6''-galloyl)-β-D-glucopyranoside **(105)** Epicatechin 3-O-gallate **(106)**

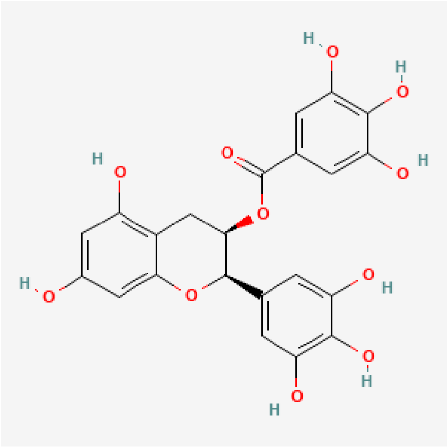

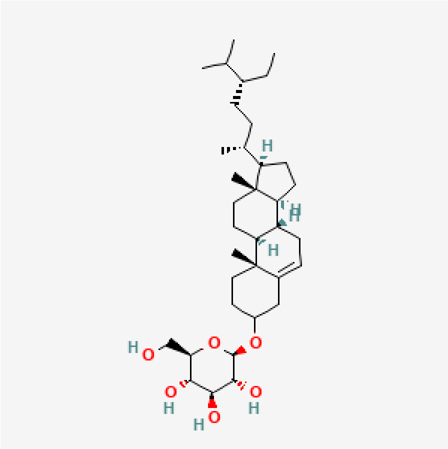

Epigallocatechin-3-O-gallate **(107)** β-Sitosterol glucoside **(109)**

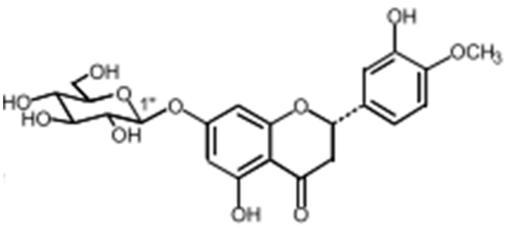

Hesperetin 3′-O-β-D-glucopyranoside **(108)**

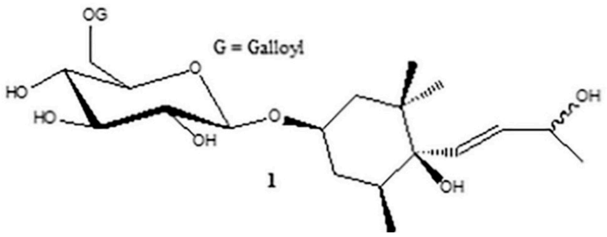

(3R*,5S*,6R*,7E,9ξ)-7-Megastigmene-3,6,9-triol-3-O-β-D-(6′-O-galloyl)glucopyranoside **(110)**

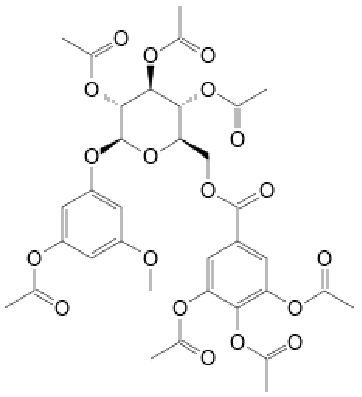

1-O-β-D-(6′-O-galloyl)-glucopyranosyl-3-methoxy-5-hydroxybenzene **(111)**

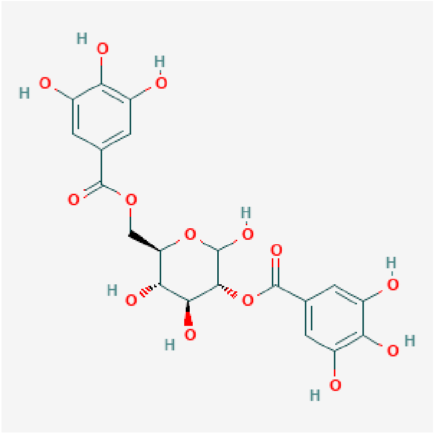

2,6-Di-O-galloyl-β-D-glucose **(112)**

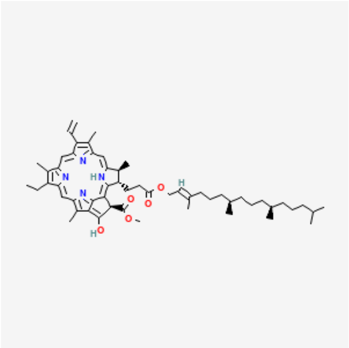

Phaeophytin A **(113)**

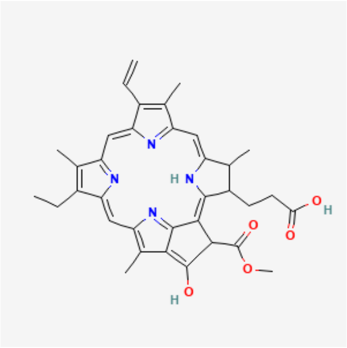

Phaeophorbide-a **(114)**

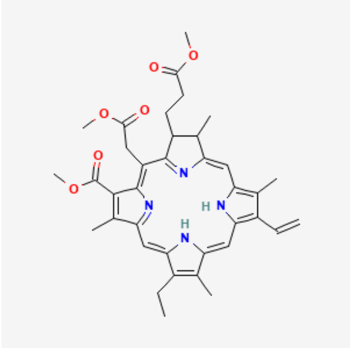

Chlorine e_6 trimethyl ester **(115)**

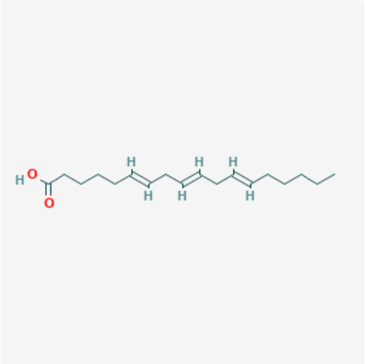

Octadecatrienoic acid **(116)**

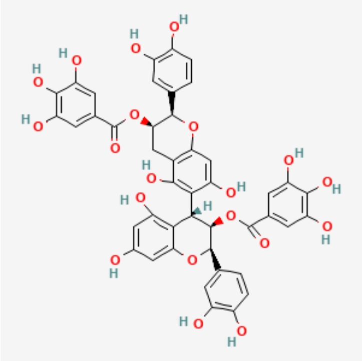

Procyanidin B5 3,3′-di-O-gallate **(117)**

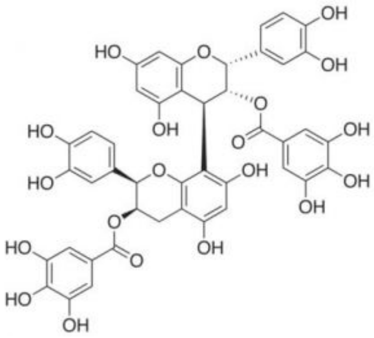

Procyanidin B2 3,3′-di-O-gallate **(118)**
